# Genetic variants associated with systemic inflammatory disease associate with temporomandibular symptoms with or without periodontitis

**DOI:** 10.1371/journal.pone.0328855

**Published:** 2026-03-25

**Authors:** Courtney Lucas, Dylan Baxter, Kathleen Deeley, Nilesh Shah, Antonio Pugliano, Renato Silva, Ariadne Letra, Mariana Bezamat, Alejandro Almarza, Juan Taboas, Alexandre R. Vieira

**Affiliations:** 1 Department of Oral and Craniofacial Sciences, University of Pittsburgh School of Dental Medicine, Pittsburgh, Pennsylvania, United States of America; 2 Center for Craniofacial Regeneration, University of Pittsburgh School of Dental Medicine, Pittsburgh, Pennsylvania, United States of America; 3 Office of Public Safety and Emergency Management, Department of Environmental Health and Safety, University of Pittsburgh, Pittsburgh, Pennsylvania, United States of America; 4 Department of Dental Public Health, University of Pittsburgh School of Dental Medicine, Pittsburgh, Pennsylvania, United States of America; 5 Center for Craniofacial Genetics, University of Pittsburgh School of Dental Medicine, Pittsburgh, Pennsylvania, United States of America; 6 Department of Endodontics, University of Pittsburgh School of Dental Medicine, Pittsburgh, Pennsylvania, United States of America; 7 East Carolina University School of Dental Medicine, Greenville, North Carolina, United States of America; Ohio State University, UNITED STATES OF AMERICA

## Abstract

**Introduction:**

Associations of genetic polymorphisms reported to play a role in systemic inflammatory diseases may serve as proxies to assess predisposition for oral diseases, as well as identify biomarkers to support preventive measures and targeted therapies. Our goal was to assess if genetic variants previously associated with systemic inflammation are associated with temporomandibular symptoms (TMS).

**Methods:**

We queried repository records to identify phenotypes (TMS and periodontitis) and systemic inflammatory diseases (asthma, obesity, rheumatoid arthritis/autoimmune disease, and type II diabetes mellitus; singular group based upon their shared inflammatory characteristic). Combinations of with and/or without systemic disease, TMS, and PD formed four groups. Single nucleotide variants (SNVs) in 15 genes (*ADAM10, AQP5, AXIN2, BRINP3, CA9, GSK3B, IL10, IL17A,IL1B, IL4, MMP2, MMP9, MYO1H, TGFB1, WNT11*) were selected for TaqMan chemistry genotyping (genotypic/addictive and allelic association tests) to identify associations between each SNP and phenotypes of interest using gPLINK. Bonferroni correction was applied (α = 0.001) to denote statistical significance. Logistic regression analyses were conducted to identify associations between systemic and dental disease phenotypes.

**Results:**

Associations were observed between SNPs in *MMP9* with systemic disease phenotypes (asthma, obesity, rheumatoid arthritis/autoimmune disease, and type II diabetes mellitus) without oral disease phenotypes (TMS-, PD-) (p = 0.00004). The same systemic disease phenotypes with signs and symptoms of TMS (TMS + , PD-) were associated with SNPs in *AXIN2* and *MMP9* (p = 0.0001 and p = 0.000009, respectively) *MMP9* was associated with the systemic disease phenotypes in the presence of periodontal disease, without TMS (TMS-, PD+) (p = 0.000008). An allelic association was found between the SNP in *AXIN2* with the systemic disease phenotypes including TMS positive phenotypes (p = 0.0005). No assocations were found between all systemic and oral disease phenotypes after controlling for age and sex at birth.

**Conclusion:**

This study showed that SNPs associated with systemic inflammation were also associated with oral diseases. These SNPs may be considered additional markers of oral disease.

## Introduction

Poor oral health continues to be a reason for developing health policies that protect those most susceptible in populations and enhance preventive strategies. Since the most common of these conditions (i.e., dental caries and periodontitis) are bacteria-mediated afflictions, fluoride-based interventions and oral hygiene-based preventive strategies are justified [1]. Through proper management of diet, maintaining an oral health regimen (routine dental appointments, flossing, brushing), the cessation of risk factors such as smoking, studies have shown a significant reduction in oral health related inflammation [2,3]. Despite the use of preventive care, some individuals still experience issues related to chronic inflammation and their related impact on oral health. Research suggests an additional underlying cause may be attributed to predisposition based upon genetic alterations or variants [2]. When this occurs, the subset of the population is at an increased risk of experiencing inflammatory disease. As a result, research aims to better understand genetic markers of inflammatory systemic disease and dental inflammatory disease phenotypes, for use as biomarkers and targeted therapies or treatments [4–7].

Furthermore, individuals affected by these common conditions usually develop additional health problems. Symptoms for these distinct conditions can be similar, such as developing discomfort, pain, and inflammation. Overtime, the chronic character of dental caries and periodontitis creating a chronic inflammatory state can lead to inadequate amino acid supply, which lead to reduced protein synthesis and degradation [1]. With that concept in mind, we believe that certain pathways will be associated with multiple conditions, for instance, pathways related to inflammatory responses. We have proposed that a more sophisticated clinical description of the phenotype may be a powerful tool for the identification of contributors to disease. Instead of selecting subjects that experience a particular condition, we characterize multiple conditions in the subjects selected to study and create more discreet groups based on presenting combinations of afflictions [8–10]. This approach has allowed for the identification of genetic contributors and other risk factors that were otherwise missed by the traditional phenotypic definitions of choosing one condition and an affected/nonaffected status in a case-control design.

Another group of conditions that is common is temporomandibular joint disorders (TMDs), which are associated with pain and dysfunction in the jaw joints and the muscles that control jaw movement [10]. Genetic associations of polymorphisms reported to play a role in inflammatory diseases may serve as proxies to assess predisposition risk assessments for other diseases, as well as biomarkers to support preventive measures and targeted therapies. Additional and comprehensive background information is presented as supplemental material (S1 Appendix). We hypothesized that genetic variants previously associated with systemic inflammatory conditions may influence temporomandibular disorders (TMDs) and possibly periodontitis. This work aimed to investigate the associations of polymorphisms in genes related to systemic inflammatory conditions in temporomandibular disorder symptoms with or without periodontitis, to identify additional markers of oral inflammation.

## Materials and methods

First, we focused on identifying genes previously associated with systemic and/or oral disease related inflammatory conditions. All SNVs (single nucleotide variants) selected for the study were identified through a literature review and from unpublished studies in our laboratory based on the following criteria: biological relevance (i.e., selection of a gene found to be relevant in disease phenotypes), global allele frequency of ~50% (to ensure a higher frequency within the study population), and the availability of on-demand SNP assays through the approved vendor (ThermoFisher Scientific) to eliminate the need for customization.

Forty SNVs were originally identified based on these criteria, of which 15 SNVs reported to be involved in both inflammatory systemic disease and/or oral disease were prioritized. Additional rationale for SNP selection and supporting information [11] is provided in Table 1.

**Table 1:** SNVs in genes selected for this study [12–36].

SNP/Base Pair	Gene/Chromosome	Name	Function	Association	Variant Type
rs10850110/109386921	*MYO1H/12*	Myosin 1H	Protein coding gene for the myosin complex	Craniofacial skeletal patterns	Intron
rs1342913/190151895	*BRINP3/1*	BMP/Retinoic Acid Inducible Neural Specific 3	Protein coding gene of cancers; polymorphisms increase vascular inflammation	Plays a role in cancer, skeleton phenotypes, growth/size/body region phenotype	Intron
rs243847/55490086	*MMP2/16*	Matrix Metalloproteinase 2	Encodes gelatinase protein (type 4 collagenase) affecting fibronectin II to bind denatured collagen (4 and 5) and elastin.	Plays a major role in cancer metastasis via cleavage of signal transduction involved ECM molecules. Mediates a pathway (CHUK/IKKA proteolysis) to trigger a primary innate immune response via proinflammatory pathways (NF-kappaB, NFAT and IRF)	Intron
rs17577/46014472	*MMP9/20*	Matrix Metalloproteinase 9	Paralog of MMP2; crucial role in ECM proteolysis and leukocyte migration. Potential involvement with osteoclastic bone resorption (cleaves collagen type 4 and 5)	Plays a major role in cancer metastasis via cleavage of signal transduction involved ECM molecules. Degradation of ECM during development and tissue remodeling; suggested role in tumor-associated tissue remodeling	Missense (G > A) Functional Consequence: non coding transcript, missense, coding sequence
rs653765/58749813	*ADAM10/15*	A Disintegrin and Metalloproteinase-10	Cell surface protein allowing for potential adhesion & protease domains (unique feature)	Family of proteins that exhibit a metalloproteinase domain play a pivotal role in the pathogenesis of tumor growth & metastasis	2KB upstream
rs2070874/132674018	*IL4/5*	Interleukin 4	The protein is a pleiotropic cytokine triggered by T cell activation.	Role in immune regulatory signal mediation, tissue repair, counteracts type 1 cytokines (proinflammatory), allergic airway inflammation, and is highly expressed in initiated immune responses as a result of chronic conditions.	5ʹ UTR
rs1554286/206770888	*IL10/1*	Interleukin 10	Encodes pleiotropic cytokine protein responsible for enhancing antibody production, proliferation, and B cell survival; mutations associated with heightened viral infections and rheumatoid arthritis.	Significant role in immunoregulation, inflammation, & rheumatoid arthritis (via mutation).	Intron
rs3748067/52190541	*IL17A/6*	Interleukin 17A	Encodes proinflammatory cytokine; role in host defense, immune modulation (auto immune/inflammatory disorders), tissue repair, cell trafficking, and cancer development/progression.	Significant role in immunomodulation, cancer metastasis, and chronic inflammatory diseases.	3ʹ UTR
rs1143634/112832813	*IL1-B/2*	Interleukin 1 Beta	Interleukin 1 cytokine family that are produced by activated macrophages (serves as a proprotein).	Major mediator of immune response, proliferation of cells, differentiation, & apoptosis.	Synonymous
rs2071676/35674056	*CA9/9*	Carbonic anhydrase 9	Transmembrane protein that serves as 1 of 2 tumor-associated CA isoenzymes; involved in cell proliferation. Catalyzes the hydration of CO2 to form HCO3- & H+ (reversible).	Established prognostic factor in Oral Squamous Cell Carcinoma(OSCC) (highly expressed in oral cancer metastasis). Expressed in hypoxic states & oncogenic alterations.	Missense (G > A)
rs2241715/41350981	*TGFβ1/19*	Transforming Growth Factor Beta 1	Multifaceted protein coding gene linked to fibrinogenesis/proliferation of fibroblasts, immunodeficiency, autoimmune disorders, bone remodeling (triggers osteoblastic activity), promotion of T-helper cells and/or regulatory T-cell differentiation and encourage sustained collagen production via CREB3L1 activation.	Abnormal TGFb1 linked to TMJ-OA like phenotypes/signs closely associated with TMD; rationale supported by the function	Intron
rs3736309/49964271	*AQP5/12*	Aquaporin 5	Promotes tumor migration, cell growth (volume, regulation, protrusion) and metastasis.	Role in oral SCC, overexpression in metastasis, secretory maintenance	Intron
rs1533767/76194756	*WNT11/11*	Wnt Family Member 11	Multifaceted function encoding secreted signaling proteins related to oncogenesis, developmental stages (e.g., skeletal, tissue)	Relation to oncogenesis, developmental alterations to the skeletal and cartilaginous structures	Synonymous
rs3923087/65553143	*AXIN2/17*	Axis inhibition protein 2	Regulation of beta-catenin in Wnt (neuronal development and pain)	Close association with Wnt, immunoexpression in the articular cartilage of TMJ (TMJ OA), and inflammatory pain	Intron
rs9879992/119993874	*GSK3B/3*	Glycogen Synthase Kinase 3 Beta	Encodes protein serine-threonine kinase which is a negative regulator glucose homeostasis involved in inflammation, ER-stress, apoptotic pathways.	Association with inflammation, cellular division, and proliferation.	Intron

### Dental registry and DNA repository (DRDR)

The Dental Registry and DNA Repository (DRDR) at the University of Pittsburgh serves as a vast source of data-banked biological samples and profiles accessible by approved investigators and students for educational and research purposes. The DRDR includes a self-reporting medical history option to provide clinicians with an established medical history per participant. Prior to the application of parameters set forth by this study, the repository in total, was comprised of 6,873 volunteer participants, with a mean age of 42.25 years old and a range of 2.5 to 97 years old. These subjects started to be recruited 9/11/2006 and the last subject recruited for the purpose of this study was in 6/30/2022. Of the 6,873, females comprised 52.37% (3,598) and males represented 47.63% (3,272) as defined as sex at birth; 3 records did not report sex at birth. Under the University of Pittsburgh Institutional Review Board (IRB) approval number 0606091, this valuable source of biological information has aided countless dental related study contributions over the years. The repository allows for the collection of human biological samples (saliva), oral health assessments and records, and readily available DNA profiles extracted via experimentation (DNA extraction assays).

### Participant record selection

The identification of qualified DRDR participant data for this study was completed through keyword filter searches within the databases for oral disease clinical diagnosis determinations, and/or self-reported diagnosis of asthma, obesity (BMI > 29), type II diabetes mellitus, and rheumatoid arthritis/autoimmune disease (Tables 2 and 3). These had additional information from medical consultations and medication history recorded in our database. Our search included information obtained via filtered headers [e.g., periodontitis yes/no [37]], and deidentified medical assessment notes provided by the dental practitioner and/or student dental care provider upon clinical evaluation. The classification of the signs and symptoms of temporomandibular disorder (TMS) was conducted by utilizing established terminology associated with the condition, such as, “discomfort and pain associated with pop, click, and/or clenching” [10] (S1 Appendix). In brief, periodontitis yes meant that at least one tooth with sites of clinical attachment loss of 4 millimeters and probing depth of 4 millimeters or deeper in the arch. Radiographs were available in all cases. Cases of gingivitis were not included. TMS yes meant having on record an accompanniyng description of any signs and symptoms in the TMJ.

**Table 2:** Details of DRDR participant records used in this study.

Disease Phenotypes	Characteristic	Status	Frequency (total N = 6870)	Percentage (%)
**Systemic** **Inflammatory** **Disease** **Phenotype**	Asthma	Negative	5921	86.19
		Positive	949	13.81
	Obesity (BMI > 29)	Negative	5356	77.96
		Positive	1514	22.04
	Rheumatoid Arthritis/ Autoimmune Disease	Negative	6694	97.44
		Positive	176	2.56
	Type II Diabetes Mellitus	Negative	6272	91.3
		Positive	598	8.7
**Oral Health/ Dental Disease Phenotypes**	Periodontitis	Negative	5692	82.85
		Positive	1178	17.15
	Temporomandibular Symptoms (TMS)	Negative	6030	87.77
		Positive	840	12.23

**Table 3:** Study demographics per systemic and oral disease status.

Disease Phenotype	Group 1	Group 2	Group 3	Group 4	Total	p-value
	TMS - & PD -	TMS + & PD -	TMS - & PD +	TMS + & PD +		
Rheumatoid Arthritis/Autoimmune Disease	15	6	4	3	28	0.1
Obesity (BMI > 29)	333	131	329	148	941	0.000
Asthma	163	105	105	81	454	0.000
Type II Diabetes Mellitus	74	32	56	40	202	0.57
**Total**	**585**	**274**	**494**	**272**	**1625**	

*TMS=Temporomandibular symptoms; PD=Periodontitis.

Applied filters for TMS were evaluated by multiple trained oral health experts to implement a rigorous screening protocol prior to participant record classification, as it is not a hard coded header within the DRDR for selection (keyword filters and manual review) (S1 Appendix). The comparison group was selected manually, however, they were in accordance with the following criteria: negative for all four disease phenotypes, negative for TMS, negative for periodontal disease, age greater than or equal to 18 years old. An attempt was made to include a minimum of two male individuals per ethnic group and two female individuals per ethnic group for each age group 18–30, 31–40, 41–50, 51–60, and 61 + years old). This study included 1,717 participants [experimental group N = 1,625; comparison group N = 92 (50% male, 50% female; minimum age of 18 years old and maximum age of 80.9 years old (mean age was 45.6 years old)] from the DRDR, representing a segment of the DRDR’s total of approximately 7,000 records.

The sourced information was organized in Microsoft Excel based upon disease phenotype or comparison group, sex, and age for assessment. Participants presenting with a comorbid condition limited to one of the four disease phenotypes were excluded from the study, as the impact of a secondary or tertiary inflammatory conditions upon genetic associations are not yet fully understood. All four inflammatory disease phenotypes were combined to form one group based upon their inflammatory characteristic to decrease heterogeneity and increase statistical power. This group will hereby be referred to as the systemic disease phenotype (Table 2). Individuals registered with the DRDR but were lacking significant details to justify study eligibility were excluded. A minimum age requirement of 18 years old was imposed to exclude child and adolescent periodontal disease, and due to the average age of disease onset for TMS (≥20 years old) and periodontal disease (≥20 years old; peak onset is 30–40 years old) [38,39].

### DNA extraction from whole saliva

Human saliva samples were processed via DNA extraction, deidentified, and data banked within the DRDR. For whole saliva samples that had not been processed for DNA extraction (n = 36), they were prepared in accordance with the Garbieri 2-day Human DNA extraction protocol for whole or fresh saliva stored for 0, 3, 6, or 12 months [40] (S1 Appendix). All saliva samples were processed to measure and record dsDNA concentration (ng/µl) using the Thermo Scientific NanoDrop One spectrophotometer; a dilution of 2 ng/µl was used for this study.

### Genotyping

Genotyping reaction plates were set up to 3 µl final reaction volumes consisting of 1µl diluted DNA and 2 µl of TaqMan reaction mix (S1 Appendix).

### End point analysis

Post PCR completion in the thermocycler, genotypes were read in the Applied Biosystems by Life Technologies QuantStudio 6 Flex Real-Time (RT) PCR system and organized per systemic inflammatory disease with and/or without an oral disease phenotype (Table 3). Undetermined genotypes were evaluated and calls were made based on the allelic discrimination plots if deemed appropriate and by two independent laboratory members.

### Data analyses

Data analyses were completed using gPLINK (http://pngu.mgh.harvard.edu/purcell/plink/) and STATA 19 (Stata Corp LCC, College Station, Texas, USA). Associations were tested by comparing allele frequencies of each group always against the comparison group.

Bonferroni correction was applied to control for multiple testing and reduce the possibility of type I error [41], considering the number of groups and tests performed. An alpha = 0.001 was used to denote statistical significance as denoted by:

(Number of disease phenotypes) x (number of SNPs) = y
(3) x (15) =45 = y
(0.05)/ (y) = Bonferroni correction alpha threshold = 0.001

### Hardy-Weinberg Equilibrium

Analysis of deviation from Hardy-Weinberg Equilibrium (HWE) was performed in gPLINK (S1 Appendix) to evaluate and quantify both the homozygous and heterozygous variants due to their allele frequency in non-evolving general populations [42]. Comparison group HWE results below the alpha threshold of 0.05 were removed (S1 Appendix).

### Genotypic and allelic association tests

Values below reported as not available (NA) by gPLINK were entered into a 2x5 Fisher’s Exact SISA calculator to determine the Chi-square (X^2^), degrees of freedom, and p-value. Chi-square cells with zero value were included in the analysis (selected “including empty -zero- cell”).

gPLINK, analytical software, may be used by researchers to complete-linkage hierarchical clustering to assess genotypic and allelic frequencies and associations with the use of SNP data [43]. Utilizing the genotyping data produced, gPLINK ped files were created per evaluation group, and one map file was created to be ran in gPLINK with a confidence interval (CI) of 95% for genotypic/addictive and allelic association tests, allelic association, and Hardy-Weinberg Equilibrium (HWE) tests [43].

### Logistic regression

Co-variates in this study were age and sex at birth (male or female). Co-variates were selected to examine the dependent variables (systemic disease phenotype (group of 4 inflammatory diseases) and oral disease phenotypes (independent variables; + /- TMS and/or periodontal disease) which have influence upon the independent co-variate variables. The use of co-variates is to establish if a relationship exists between the phenotypes [systemic (asthma, obesity, type II diabetes, and rheumatoid arthritis/autoimmune disease) and oral disease/dental (TMS and/or PD)] and both age and sex.

Statistical analysis was completed using STATA that provides a user-friendly interface that allows for the completion of a variety of statistical tests. STATA was utilized to complete logistic regression of the single, independent variable (disease phenotype) and the dependent variables: age (continuous variable) and sex (categorical variable) to identify influence/a relationship.

## Results

Of the fifteen SNPs tested, three (*IL10* rs1554286, *ADAM10* rs653765, and *MMP2* rs243847) showed deviation of HWE in the comparison group and were excluded from further analyses (S1 Appendix).

### Genotypic and allelic association tests

Group 1 was represented by the individuals with the systemic disease phenotype (individually, a person was diagnosed with only 1 of 4 disease phenotypes listed who met the study inclusion criteria for age (≥ 18 years old); they were then placed into an aggregate group representing 1 overall disease phenotype) who are negative for both TMS (-) and PD (-). Group 2 was positive for the systemic disease phenotype, positive for TMS signs and symptoms, and negative for periodontal disease. Group 3 was positive for the systemic disease phenotype, negative for TMS signs and symptoms, and positive for periodontal disease. Group 4 represented a group who are positive for the systemic disease phenotype, TMS, and periodontal disease. These four groups were each compared to a pre-defined comparison group of 92 individuals that did not have a diagnosis of any of the inflammatory conditions listed in Table 3. Table 4 summarizes the significant results and full genotypic/addictive and/or allelic association tests in the study groups are listed in the supplemental material (S1 Appendix).

**Table 4:** Summary of the significant results.

Group	Gene	Genetic Variant	Genetic Model	Individuals Affected	Individuals Unaffected	p-value
1	*MMP9*	rs17577	Genotype	9/102/285	9/25/40	0.00004
			Allelic	120/672	43/105	0.00004
			Recessive	9/387	9/65	0.00005
2	*AXIN2*	rs3923087	Allelic	128/276	75/77	0.0001
			Dominant	99/103	56/20	0.0002
			Genotypic	29/70/103	19/37/20	0.0009
	*MMP9*	rs17577	Allelic	56/372	43/105	0.000009
			Genotypic	4/48/162	9/25/40	0.00006
			Dominant	52/162	34/40	0.00045
3	*MMP9*	rs17577	Genotypic	7/100/277	9/25/40	0.000008
			Recessive	7/377	9/65	0.000009
			Allelic	114/654	43/105	0.00002
4	*AXIN2*	rs3923087	Allelic	147/291	75/77	0.0005

Notes: Allelic model assumes the risk/effect increases linearly with each copy of the less common allele. The Dominant model assumes a single copy of the less common allele is sufficient to produce full phenotypic effect. The Recessive model assumes the phenotypic effect is only preent in the individual crries two copies of the less common allele. The Genotype model is a multi-degree-of-freedom test that makes no assumptions about the mode of inheritance, testing each genotype separately.

### Logistic regression

Logistic regression analysis was carried out to identify associations between the systemic health condition (yes/no) and TMS (Tables 5 and 6).

**Table 5:** TMS Logistic Regression Model 1. Command executed ➔ xi:logit DiseasePhenotype i.TMS,or.

TMS Disease Phenotype	Odds Ratio	Standard Error	Z-score	P > |z|	[95% conf. interval]	
TMD	**1.026383**	0.0641427	0.42	**0.677**	0.9080598	1.160124

**Table 6:** TMS Logistic Regression Model 2. Command executed ➔ xi:logit Disease Phenotype i.TMS Age i.Sex,or.

TMS Disease Phenotype	Odds Ratio	Standard Error	Z-score	P > |z|	[95% conf. interval]	
TMS	**0.9799615**	0.0624367	−0.32	**0.75**	0.8649203	1.110304
Age	1.020393	0.0016908	12.18	0	1.017084	1.023712
Sex	0.7405437	0.0447352	−4.97	0	0.6578559	0.8336246

Model 1 depicts the logistic regression of the systemic disease phenotype aggregate group [all four systemic health conditions (obesity, asthma, type II diabetes mellitus, and rheumatoid arthritis/autoimmune disease)] in relation to temporomandibular symptoms (TMS) (Table 5). Model 2 is the execution of logistic regression of the systemic health conditions and TMS with potential confounding factors age and sex (male or female) (Table 6). The outcome for both models established there was no association between TMS and systemic disease phenotype, as well as there is no association under the consideration of potential confounding factors.

## Discussion

Associations of genetic polymorphisms reported to play a role in systemic inflammatory diseases (asthma, obesity, type II diabetes, and rheumatoid arthritis/autoimmune disease) may serve as proxies to assess predisposition risk for oral diseases (phenotypes: temporomandibular diseases and/or periodontal disease), as well as identify biomarkers to support preventive measures and targeted therapies [44–46]. We hypothesized that genetic variants previously associated with systemic inflammatory conditions may influence temporomandibular disorders (TMDs) and/or periodontal disease predisposition. To test the hypothesis, our study focused on identifying associations of polymorphisms in genes related to systemic inflammatory conditions with and without temporomandibular disorders and periodontal disease to identify additional markers of oral inflammation.

Experiments conducted sought to identify the associations between polymorphisms in genes related to systemic inflammatory conditions with obesity, type II diabetes mellitus, rheumatoid arthritis/autoimmune disease, and asthma to identify additional markers of oral inflammation. SNPs investigated included *IL17A, AXIN2, MMP9*, *BRINP3*, *IL1B*, *GSK3B*, *IL4*, *CA9*, *WNT11*, *MYO1H*, *AQP5*, *TGFB1*, *IL10*, *ADAM10*, and *MMP2* genes. Genotypic/addictive, recessive, and allelic associations for the SNP in the *MMP9* gene, were identified with the systemic inflammatory disease phenotype (asthma, obesity, rheumatoid arthritis/autoimmune disease, and type II diabetes mellitus).

We also investigated the associations of polymorphisms in genes related to systemic inflammatory conditions (obesity, type II diabetes mellitus, rheumatoid arthritis/autoimmune disease, and asthma) considering two phenotypes: TMS and/or periodontitis. The same fifteen SNPs were tested. For Group 2, the systemic disease phenotype with signs and symptoms of TMS (TMS + , PD-) was found to be TMS (TMS genotypically and allelically associated with the SNP in *AXIN2*, as well as *MMP9*. Group 3 showed genotypic and allelic associations between the systemic disease phenotype in the presence of periodontitis, without -, PD+). Group 4 identified an allelic association for SNPs in *AXIN2* with the systemic disease phenotype including both oral disease phenotypes (TMS + , PD+). Logistic regression analyses did not identify any significant associations between the investigated SNPs with TMS or PD after controlling for age or sex at birth.

The *MMP9* gene, also referred to as gelatinase B and encodes matrix metalloproteinase-9, is a zinc-dependent endoproteinases that assists extracellular matrix (ECM) degradation, and therefore is implicated in physiologic and pathological bone remodeling via the promotion of osteoclastic activity (resulting in studies evaluating use of the gene as an osteosarcoma biomarker), as well as wound healing/repair [47,48]. Upon dysregulation, *MMPs* are prone to trigger the destruction of the ECM and/or surrounding tissue or impair wound healing [47]. *MMP*s have been extensively studied with regards to their roles in a myriad of inflammatory diseases. For example, *MMP9* has been shown to be upregulated during a neuroinflammatory response, as encoding effector molecules of the immune-inflammatory system [49]. During respiratory/pulmonary conditions such as chronic obstructive pulmonary disease (COPD) and asthma, *MMP9* has been shown to modulate other biological factors contributing to pulmonary tissue destruction [47]. In contrast, MMP levels in coronary artery disease patients were not attributable to genetic variants but rather are due to metabolic disorders [50].

The *MMP9* SNP associated in the present study, rs17577 (0.19 G > A), is a non-synonymous SNP that results in a singular amino acid substitution of an arginine to a glutamine (Arg668Gln) at position 668 of the protein structure; its function is predicted as benign. Our study has identified genotypic and allelic associations between *MMP9* rs17577 with the systemic inflammatory disease phenotype (obesity, type II diabetes mellitus, rheumatoid arthritis/autoimmune disease, and asthma). Associations were also found between the SNP and systemic disease with or without oral disease phenotypes: Group 2 TMS + /PD- and Group 3 TMS-/PD + .

In a case control study of asthma risk in Mexican pediatric patients, *MMP9* rs17577 had a significant association with asthma risk (OR=2.07, 95% CI 1.29–3.30, P = 0.001); it also showed association with atopic asthma [35]. Further, in a meta-analysis, three *MMP9* SNPs (rs17577, rs3918242, rs17576) were reported as significantly associated with asthma risk. Of note, *MMP9* rs17577, displayed significant associations under dominant, homozygote genotype, and allelic genetic models, thus the researchers concluded the polymorphism is an appropriate marker of asthma susceptibility [36]. While our study used an adult population, these association findings with *MMP9* rs17577 in both children and adults [36] reinforce the evidence for this SNV’s role in systemic and oral phenotypes.

A polymorphism in Axis Inhibition Protein 2 (*AXIN2),* rs3923087 (0.45 C > T), also showed significant association with the systemic health conditions and oral diseases investigated (TMS and PD): genotypic and allelic associations were found for group 2 (TMS + , PD-), along with an allelic association in group 4 (TMS + , PD+). *AXIN2* is a protein coding gene and a negative regulator of the Wnt-Beta catenin pathway which plays important roles during embryogenesis and tumorigenesis. Mutations in *AXIN2 (e.g.,* Arg656Stop nonsense mutation) were shown to predispose affected individuals to conditions such as colorectal cancer and tooth agenesis (congenitally missing teeth) due to dysregulation of WNT pathway signaling activity [51]. Similarly, mutations in *AXIN2* have been associated with gastric cancer [52] and *AXIN2* SNVs have also been linked to tooth loss in individuals diagnosed with cancer [53]. In a recent study assessing the relationship between tooth loss/edentulism and oral squamous cell carcinoma (OSCC), *AXIN2* rs3923087 was statistically associated with OSCC in individuals with tooth loss (OR=0.58; 95% CI:0.34–0.97). Specifically, the C allele was less frequent in individuals with tooth loss diagnosed with OSCC (OR = 0.42) in comparison to controls [54]. These findings are intriguing and warrant additional studies that may help us understand the role of genetic variation potentially linking systemic and oral disease phenotypes.

In a study focused on temporomandibular joint disorders (TMD) investigating how AXIN2 modulates Wnt signaling pathway activity, the pathway was activated using polarized macrophages to drive inflammation within the temporomandibular joint (TMJ). Results showed that *AXIN2* was the target of two microRNA targets resulting in an inhibitory effect in signaling. The resulting inhibition then triggered pathway activation, and an influx of inflammatory response occurred in the joint space [31]. These findings identified *AXIN2* as a key regulator in the TMJ inflammatory process that has the potential to be manipulated to induce a response. While this study did not look at *AXIN2* polymorphisms, it shows that regulation of the gene via additional genetic regulatory mechanisms may also contribute to oral disease phenotypes.

Based on these results, we can propose that variants in *AXIN2* and *MMP9* can be used as genomic markers to identify individuals at risk of certain systemic conditions (asthma, obesity, type 2 diabetes, and arthritis) that will also potentially develop oral conditions (TMS and periodontitis). Future experiments should be carried out with comprehensive and sophisticated clinical assessments that will define the presence of multiple conditions in an individual and have as a comparison, individuals not experiencing any of the conditions. We firmly believe that it is time that we start favoring studying the combination of multiple conditions in the same people rather than one disease at a time.

In summary, our study showed the associations of SNVs in two genes (*AXIN2* rs3923087, and *MMP9* rs17577) with the systemic and/or oral disease phenotypes investigated. The lack of association with the additional 13 SNPs investigated may be attributed to numerous reasons that may include, the sample population size, the single SNV per gene selected for our study, and reporting practices of disease diagnoses.

## Study limitations

### Medical history reporting

The DRDR platform is primarily geared towards the compilation of dental health information as a separate entity from regional medical record databases not allowing for a holistic, comprehensive overview of the participants’ medical and oral health history. Medical history information provided upon DRDR study intake is reliant upon self-reporting practices, therefore, there is an inherent risk of incomplete reporting, or missing data. The National Academies of Sciences, Engineering, and Medicine (NASEM) TMD consensus study report presents TMD as an umbrella disorder that embodies thirty health conditions involving the joint and it’s supportive structures [55]. As such, the self-reporting practice for medical history and a lack of standardized options available to further classify the appropriate TMD sub-group is a limitation of our study.

### SNV selection

Our study selected one SNV per gene, totaling fifteen SNVs in genes. A SNV is a single nucleotide variant that contributes to singular point mutation found within a specific segment of a sequence. Although associations may or may not be found, it’s important to note that the outcome cannot rule out the possibility for another SNP in that gene to yield a different outcome.

### Geographic location and study population

Geographic location of sample collection is limited to the University of Pittsburgh School of Dental Medicine in Pittsburgh, Pennsylvania, a city with a “2021 population estimation of 193,000 individuals that is comprised of 63.8% White (non-Hispanic), of which is 2.81 times more than any other racial or ethnic group in the city [22.7% Black or African American (Non-Hispanic), 5.55% Asian, and 3.96% Multiracial (non-Hispanic)] [56]. A proportion that is mirrored within the DRDR’s study population of 7,000 participant records. Geographically, Pittsburgh is considered an isolated city in Western Pennsylvania (west of the Appalachian mountains) in relation to the distance to the nearest metropolitan city. Despite serving the area as a leader in healthcare and a hub for colleges, Pittsburgh grossly lacks ethnic and racial diversity, which is evident within and study participant pool. This lack of diversity and limited study population may impact the observed trends; if the study population was expanded and represented racial/ethnic diversity aligned with the US population, it would increase the validity of the data captured and serve as a more accurate representation.

The lack of sex-specific analysis is a limitation of this study; sub-groups of more than 100 participant records are ideal, however, the modest size of the study population would have yielded smaller sub-groups (e.g., participant records for females (at birth) 18 years and above with asthma) of a lesser statistical power. As a result, we did not conduct a sex-specific analysis to maintain reliable statistical data. However, we included sex as a covariate in the adjusted analysis.

### Multifactorial contributions

Inflammatory diseases such as asthma, obesity, type II diabetes, rheumatoid arthritis/autoimmune disease, temporomandibular disorders, and periodontal disease are all considered to be multifactorial conditions. By definition, multifactorial conditions/diseases are the result of numerous genetic and/or environmental factors [57]. The purpose of this study was to identify genetic polymorphisms in inflammatory disease to elucidate additional markers of inflammation. As the study focused solely upon the genetic aspect of disease, it does not address the social/environmental contributions to disease onset or pathogenesis, therefore, providing a limited perspective.

The influence of social/environmental factors such as tobacco use, exercise/physical activity, stress, diet, sanitation practices, socio-economic status, and heavy metal/chemical exposure influence health outcomes [58–60]. An example of a social/environmental contribution not explored is the experience of heightened stress which leads to repetitive clenching and/or bruxism and prolonged temporomandibular joint (TMJ) pain experienced by the individual, attributing to a diagnosis of temporomandibular disorder (TMD). The DRDR includes self-reported information regarding tobacco use as a yes/no field, as well as the amount of use (e.g., 3 times daily). Tobacco use/smoking was not utilized for the purposes of this study, as the field is self-reported and are reliant upon participant recollection, a willingness to provide a true and accurate account of their usage, and health literacy. Research has shown that the use of self-report tools lack an ability to account for changes or context regarding the field of interest such as lifestyle activity [61]. An objective measurement is preferable (e.g., urine, blood, saliva, or hair sample for nicotine cotinine) in comparison to a self-reported measure to maintain quality of the data, therefore, tobacco use was not included. Although genetics has been shown to have a significant factor in the development of inflammatory disease, the inclusion of social/environmental contributions are needed to best provide a complete understanding of disease predisposition risk [62,63].

## Conclusion

This study found associations between *MMP9* rs17577, *AXIN2* rs3923087, and *IL-17A* rs3748067, and systemic inflammatory conditions with or without TMS or periodontitis.

## Supporting information

S1 Appendix Additional background information, methodology, and detailed results. (DOCX)
