# Supplementary material for: Genetic variants associated with systemic inflammatory disease associate with temporomandibular symptoms with or without periodontitis
Source: PLoS One. 2026 Mar 25;21(3):e0328855. doi: 10.1371/journal.pone.0328855 (PMC13016321; doi:10.1371/journal.pone.0328855)
Supplement: S1 Appendix — (DOCX) [file pone.0328855.s001.docx]

**Supplemental Materials**

**Additional Background Information**

Inflammation is a prevalent systemic condition characterized by the biological action involved in both the recruitment and activation of protective cells, in response to stimuli recognized as a potential threat (1). During the response, an influx of protective antibodies and immune cells infiltrate the area to attack the foreign stimuli. In time, the body can heal and return to its original protected state; however, in many cases, the innate and/or adaptive immune-inflammatory response is overpowered, thus not allowing the body to return to a healed, unaffected state. When this state of attack is prolonged without resolution, the body becomes hyper-inflamed, the immune system weakens, the potential for additional immunological attacks increases due to its weakened state, and typically, there are notable outcomes such as fever, pain (neurosensory agitation), swelling, neuromuscular impairment, and structural degradation (e.g., loss of cartilaginous components of joints, as seen in arthritic conditions) (2).

Inflammatory diseases are a common occurrence in adults, adolescents, and children of both the male and female sex, world-wide. Inflammatory diseases vary based upon mechanism(s) of action, complexity, and severity (i.e., acute or chronic). Acute inflammation occurs as a minute response, however, chronic inflammation is persistent and prolonged. Chronic inflammation is a heightened immunological state commonly classified as an inflammatory disease. Examples of chronic inflammatory disease includes, but is not limited to asthma, obesity, cardiovascular disease, inflammatory bowel disease (IBD), arthritis (rheumatoid, psoriatic, osteoarthritis, ankylosing spondylitis), and auto-immune diseases (e.g., sickle cell disease and lupus) (3). Studies have shown that social determinants and socioeconomic risk factors such as smoking, obesity, age, nutrition, hormonal fluctuations, financial status, chemical exposure, and stress, as well as genetics, may influence predisposition and/or progression to chronic inflammatory disease (4-6).

Research has shown that inflammation in a chronic state may drive the onset and progression of local oral disease conditions and systemic disease (7). The continuous destruction of cellular processes and structures because of chronic inflammation may have deleterious effects on health. As such, inflammation has an effect on oral disease in several ways which include the appearance of redness and swelling of the gingiva, localized orofacial pain, increased sensitivity, gingival tissue recession, the development of lesions/sores/ulcers due to the destruction or disturbance of a protective layer of cells typically serving as the outmost lining of the oral mucosa, and increased susceptibility to bacterial invasion (7).

Through proper management of diet, maintaining an oral health regimen (routine dental appointments, flossing, brushing), the cessation of risk factors such as smoking, studies have shown a significant reduction in oral health related inflammation (8,9). Despite the use of preventive care, some individuals still experience issues related to chronic inflammation and their related impact on oral health. Research suggests an additional underlying cause may be attributed to predisposition based upon genetic alterations or variants (8). When this occurs, the subset of the population is at an increased risk of experiencing inflammatory disease. As a result, research aims to better understand genetic markers of inflammatory systemic disease and dental inflammatory disease phenotypes, for use as biomarkers and targeted therapies or treatments (10-13).

Four highly prevalent systemic inflammatory disease conditions are asthma, obesity (body mass index, BMI>29), type II diabetes mellitus, and rheumatoid arthritis/autoimmune disease. Due to the heightened prevalence of disease, the four were selected for further studying as a combined disease phenotype, as all represent the study focus, an inflammatory condition.

Asthma is a multifactorial (e.g., genetic, allergic reaction, over-exhaustion, exercise-induced), chronic inflammatory condition resulting in the partial obstruction of the bronchial tube, or airway, due to an inflammatory response (14). This obstruction leads to restricted breathing and wheezing that may be treated with a medicine such as a breathing treatment, a rescue inhaler to provide an aerosolized steroid (corticosteroid), or a beta-agonist bronchodilator such as albuterol for relief (15). Approximately 262 million individuals worldwide have the disease, with the highest prevalence observed in the United States (16). Asthma is typically first observed in adolescence, however, adult onset is not uncommon, as it is influenced by numerous factors such as allergic reactions, over-exhaustion, lifestyle changes, environmental irritant exposure, exercise, and genetic mutations (17-19).

Obesity-related asthma is an underlying issue for millions of individuals worldwide, however, the pathogenesis of disease regarding the immunopathological mechanism of action is not yet fully understood (20). Genetic linkage and genome-wide association studies (GWAS) have identified associations between genetic variants in *ORMDL3 (ORMDL* Sphingolipid Biosynthesis Regulator 3; regulator of inflammasome activation during allergic exacerbation) and *GSDMB* (Gasdermin B; regulates aryl hydrocarbon receptor (AHR), inflammatory mediator release, airway remodeling) with childhood onset asthma, *IL33* (interleukin 33; aids rapid immune response, has the ability to trigger type 2 inflammatory responses) and *IL1 RL1 (*interleukin 1 receptor like 1; type 2 cytokine and chemokine production) with atopic asthma, and *TSLP (*Thymic Stromal Lymphopoietin) with protective properties against T-helper 2 cell (TH2) asthma (the primary T cells responsible for non-allergic asthma) (19). The information discovered from the studies have allowed a more comprehensive understanding of the disease mechanisms (19,21). As a result, the addition of epigenetic research, a branch of scientific study focused upon the observation of the genes as they undergo changes that modify their status without changing the DNA sequence via DNA methylation, as well as histone and microRNA (miRNA) expression have provided valuable information to the body of knowledge for asthma susceptibility within the field (19).

As asthma affects the airways, of which include the lungs, larynx, airways (trachea, bronchioles, and bronchi), throat, mouth, and nose, it is important to utilize experimental methods to appropriately evaluate asthma. Epigenome-wide association studies (EWAS) for asthma has been conducted separately on the nasal epithelium and blood, however, there are differences for each approach: nasal epithelium is a route that was easily reproduced for the testing of subjects and it depicts both the changes in DNA methylation and dysregulation of epithelial cells found within the respiratory tract, and blood EWAS depicts eosinophilic disruptions in immune cells of the blood (19). The EWAS evaluations discovered tissue-cell specificity for patterning developed during methylation (DNA) in asthma, however, the presence of common genes for both blood and epithelial tissue of the nose (*ACOT7, EPX, GJA4*, and *METTL1*) suggest that in addition, a cross-tissue epigenetic effect is also present for asthma (19).

Histone modifications are also affected by asthma, as there are post-translational modifications that affect bronchial asthma pathogenesis through histone acetylation via and deacetylation (19). Histone acetyltransferase (HAT) provides information regarding asthma status with biopsies revealing elevated activity for adults in children, while a ratio of HAT and histone deacetylase (HDAC) (HAT/HDAC) correlates to disease severity (19).

The use of microRNAs (miRNAs) have been used in epigenetic research, as they serve as the regulators of genetic expression many messenger-RNAs (mRNAs). The upregulated or downregulated expression status of miRNAs provides information relating to the impact upon disease pathogenesis. For example, an upregulation of miR-21 (makes production and proliferation contributions of eosin) and miR-126 in afflicted patients compared to the control group has been reported (19). In addition to the eosinophilic contributions of miR-21, it is also linked to repression of IL-12p35, leading to steroid insensitivity in asthma patients, which may be useful as a biomarker to identify a patient’s treatment response to steroids (19).

Studies have also reported a link between asthma and/or asthma treatments and an increased risk of dental caries, erosion of the teeth, periodontal disease, and/or the development of oral candidiasis due to repeated exposure to asthma medication and its induced xerostomia (22). As such, alternative treatments are highly sought after to reduce the chance of these adverse outcomes. Genetic polymorphisms that are identified as functional with impact to disease phenotype may provide insight into therapeutic targets or preventive measures to improve patients’ quality of life.

Obesity in adults is characterized by a body mass index (BMI) > 29 and defined as the excessive accumulation of body fat in relation to an individual’s height, weight, age, and sex. Worldwide, obesity rates continue to rise in established countries with food-secure communities (23). According to the World Health Organization, in 2022, 890 million adults over the age of 18 years old were considered obese, accounting for 16% of the world population (24). Obesity is a multifactorial condition and the etiology may include genetic predisposition (e.g., affecting variables such as how an individual stores fat and works to convert food into energy), environmental and social influences, and metabolic pathway disturbances (medical diagnoses such as hyperthyroidism; hormonal fluctuations such as thyroid, insulin, leptin), which may influence genes, the urge for caloric intake, mental gratification and stability from fullness (25). As a result, obese individuals may experience low-grade chronic inflammation with oral inflammatory manifestations (26-28). Heritable obesity has a strong genetic etiology that may be either monogenic or polygenic. Approximately eighty obesity-related syndromes have been identified, however, only a portion of the etiology has been identified (29). In total, twenty-three syndromes are genetically heterogeneous and include structural variants (e.g., insertions), albeit clinical presentations are relatively homogeneous (29).

Non-syndromic monogenic obesity is commonly characterized as either an endocrine disorder or hyperphagia (unsatiated hunger) resulting from genetic variation. Examples include leptin receptor (LEPR) mutations leading to thyroid and growth hormone dysfunction (2-3% of a population); autosomal recessive and dominant melanocortin-4 receptor (MC4R) mutations triggering increased appetite in children; MC4R missense variants in 5% of pediatric obese patients; loss of function mutations in SIM1 leading to pediatric obesity; and kinase suppressor of Ras 2 (*KSR2)* triggering hyperphagia and insulin resistance (29).

Three sizeable GWAS studies reported that 30-55% of fat distribution is attributable to genetics; within a sub-group of loci determined to be significant, the presence of sexual dimorphism was discovered (29). In addition, genetic associations with visceral adipose tissue and waist circumference (WC) were found near the gene Threonine Synthase Like 2 (*THNSL2)* only in women. The gene is linked to decreased physical activity and increased weight. The *THNSL2* associations were identified near Bardet-Biedl Syndrome 9 (*BBS9)* and CYCS pseudogene 30 (*CYCSP30),* which are regulated by the parathyroid hormone and has an influence on metabolic regulation, respectively (29). Associations with liver fat were also identified among the following genes: UDP glucuronosyltransferases (*UGT1A)* which regulates glucuronidates bilirubin*;* suppressor of cytokine signaling 2 *(SOCS2) -* the gene regulates lipid metabolism, growth hormone, and adipose tissue inflammation*;* Receptor Activity Modifying Protein 3 (*RAMP3) –* a protein coding gene associated with immunodeficiency, inflammation, and deficiency aids tumor suppression (29,30)*.*

Treatment modalities for obesity include weight loss management through the introduction of a well-managed diet (caloric deficit with balanced meals), routine exercise, medication for appetite suppression (e.g., tirzepatide, sumaglutide), and surgical options such as bariatric surgery (25). Implementation of treatment modalities work to improve symptoms of the disease over an extended period of time (long-term), as opposed to providing a quick cure, however, there are many benefits associated with adherence to a well-balanced treatment plan focused upon several aspects of disease management (e.g., exercise and mobility goals, diet, behavior (social factors), and including understanding the role of genetics in disease predisposition. The use of interventional methods to promote weight loss may lead to improved health such as a reduction in type II diabetes mellitus, cancer, and obesity-related asthma risk; decreased blood pressure which is typically elevated for obese patients, a reduction in lipids that promotes improved cholesterol (reduced fatty lipid deposition prevents clogged arteries, leading to a reduction in blood pressure), normal glycemic levels, and a reduction of or elimination of sleep apnea (25).

Non-insulin dependent diabetes mellitus (type II diabetes) is a chronic, inflammatory condition of global health concern. As of 2022, it is estimated that out of 96 million Americans, at least 1 in 3 have prediabetes and more than thirty-seven million Americans have been diagnosed with diabetes (approximately 1 in 10 individuals) (31). Of the thirty-seven million, approximately 90-95% individuals have been diagnosed with type II diabetes, making it the most prevalent type of diabetes (32). The Diabetes Atlas published by the International Diabetes Federation (IDF) estimated in 2021 that the diabetes-related direct health expenditures was around one trillion dollars, and is anticipated to exceed that amount by the year 2030, thus making it a global health crisis (31). Although the condition is commonly referred to as adult onset diabetes, children, adolescents, and adults may develop type II diabetes, which is in contrast to 20 years prior (there has been an increase in children and adolescents diagnosed with type II) (31). It has been found that in addition to increased obesity due to lifestyle and environmental factors, approximately 75% of children with type II diabetes show familial inheritance (31).

In addition to familial prevalence, specific ethnic groups are more likely to develop the disease: African American, Asian American, and Hispanic/Latinos are at a 17% increased risk of developing the condition in comparison to non-Hispanic White people who have an 8% risk. Within the United States, the Hispanic/Latino community have approximately a 50% likelihood of developing type II diabetes, and the majority will experience disease onset at a much younger age (33,34). The hereditable connections and the statistics displaying an increased risk of developing the condition within specific ethnic or racial groups provide support that genetics can be an important factor in this multifactorial, polygenic disease (34). A meta-analysis study of *KCNQ1* polymorphisms reported six significant associations (rs2237892, C allele), rs2237897 (undetermined), rs151290 (C allele), rs2283228 (A allele), rs2074196 (G allele), and rs2237895 (C allele)) between the *KCNQ1* polymorphism and type II diabetes mellitus, and may be utilized as a type II diabetes biomarker for disease susceptibility, particularly in Asian populations (35).

Adiponectin is a protein involved in insulin sensitivity through regulated insulin release from the pancreas and basal glucose (36). This mechanism combats insulin resistance, as insulin resistance yields hyperinsulinemia, a condition responsible for increased glucose, type II diabetes, and weight gain/obesity. The interplay between adiponectin and type II diabetes is associated with increased adiposity and insulin resistance. In a systematic review and meta-analysis evaluating the relationship between polymorphism in the adiponectin gene (*ADIPOQ)* and type II diabetes, an association was found with the promoter variant -11377C>G (rs266729) suggesting that this polymorphism may drive the expressed function/action of adiponectin (36). This discovery provides rationale for use as a therapeutic treatment, as the increase in adiponectin production has the potential to decrease insulin resistance, along with its anti-inflammatory and anti-atherogenic effects from the protein (36). The ability to increase the production or release of adiponectin would benefit type II diabetes mellitus patients by reducing the negative outcomes of disease.

The disease trajectory typically begins as a pre-diabetic stage marked by slightly abnormal glucose levels which may return to a normal level after consistent medical intervention (doctor’s supervision, diet, and exercise management). This stage is rarely identified by the individual, as it is typically the result of routine check-ups with their physician, therefore, it is important to abide by routine medical appointments. The onset of type II diabetes is a gradual process characterized by the pancreas’ initial inability to provide enough of the chemical hormone insulin responsible for supporting the uptake of glucose into cells to be used as energy. When the pancreas does not receive an adequate response from the cells displaying insulin resistance, the hormone becomes less effective, and the pancreas reacts by producing more insulin (hyperglycemia), creating detrimental effects such as glucose spikes, vision impairment or loss, chronic kidney disease, heart disease, prediabetes, and type II diabetes (31,33,37).

Type II diabetes has been shown to also negatively impact oral health: an increase in unprocessed glucose creates a food source for bacteria found in dental caries, resulting in expedient tooth decay and periodontal/gingival infections (9). As a result, wound healing is also delayed, leading to complex treatment plans that have a higher failure-rate, especially if the diabetes is not controlled (38). Unlike type I diabetes, type II may be undiagnosed for a significant amount of time prior to identification and treatment, thus upon detection, the patient may present with additional serious health complications (38).

Uncontrolled type II diabetes may play a critical role in the development of systemic conditions which affect the major systems: immune, circulatory, and/or nervous systems (38). Due to the role in immunological crosstalk between both adipose tissue and macrophages, as well as sites for cells necessary for proper function of immune response, more research is needed to fully understand the full mechanism in relation to its behavior in the presence of type II diabetes mellitus (39). Type II diabetes is a multifactorial condition that has polygenic triggers, and may be clinically diagnosed through the use of an ANC (absolute neutrophil count) test, fasting blood sugar test, glucose tolerance test, BMI determination, and a physical medical examination (40). Type II diabetes mellitus may negatively impact oral health, as an increase in unprocessed glucose creates a food source for bacterial plaque to form, resulting in expedient periodontal/gingival infections (9). As a result, routine oral healthcare is recommended for individuals diagnosed with the disease to provide an established regimen to prevent or treat disease-related outcomes.

Diet, exercise, medication (e.g., metformin), therapy [singular or in combination; e.g. sulphonylureas to trigger insulin production by the beta cells, alpha glucosidase inhibitors to act upon the gut to delay uptake of carbohydrates, thiazolidinediones to help activate insulin, dipeptidyl peptidase 4 (DPP-4) inhibitors, glucagon-like peptide 1 (GLP-1) agonists and sodium glucose co-transporter 2 inhibitors], smoking cessation, and weight loss are effective methods to temper the disease, however, at this time, there is no cure for the condition (31,40,41). A genetic epidemiology study found there are more than 400 genetic variants associated with type II diabetes mellitus that may be utilized as predictors of disease, in conjunction with body mass index (BMI) reporting to accurately determine risk, as genetic indicators alone are not sufficient to predict health outcomes (42). Upon diagnosis, patients may be prescribed medication to treat the symptoms of the disease. Currently, available medication only targets the symptoms of type II diabetes mellitus, therefore, there is a need to identify a promising approach to biomarkers that may be used to gauge predisposition risk, allowing clinicians to provide appropriate targeted therapies and preventive treatments (40,43).

Rheumatoid arthritis is a long-term autoimmune disease that results from the body’s painful response to chronic inflammation infiltration of the joints (44). It is typically found within the joint spaces of the hands and feet, and can result in abnormal swelling of the joints, pain, redness, joint degradation, and limited mobility dependent upon the degree of severity (44). Approximately 0.37 to 1.25% of the population worldwide has been diagnosed with rheumatoid arthritis, and it is most prevalent in women (0.75%); women in the United States experienced a 2.5% increase in incidence (45). Unfortunately, individuals afflicted with rheumatoid arthritis are more likely to experience a negative impact on life expectancy (decreased by approximately 3-10 years) (46). As a multifactorial disease, the etiology is not yet fully understood, however, risk factors for incidence include sex at birth (female), environmental factors, hormonal fluctuations, immunological disease, genetics, tobacco use, ethnicity, “rheumatoid factors, and/or anti-citrullinated protein antibodies” (45,47).

Studies researching rheumatoid arthritis susceptibility in twins and linkage analyses discovered a heritability estimate of 60% (46). The estimate was significantly comprised of Human Leukocyte Antigen class II (HLA-II) genes (30%), which is a subgroup of Major Histocompatibility Class (MHC) class II (46). In particular within HLA-II, HLA-DRβ1 which functions as a peptide presenting heterodimer that participates in the immune system, was found to have the strongest genetic association for the disease (30,46). The strength of the association classified HLA-II as a major contributor of rheumatoid arthritis susceptibility (46).

A genomics study utilizing two metagenome approaches (shotgun sequencing and metagenome-wide association study) evaluated healthy individuals and rheumatoid arthritis patients by sampling fecal, oral swabs, and saliva for testing. They found the microbiome between the two groups (healthy and diseased) displayed measurable differences, in which rheumatoid arthritis patients can be identified based upon their microbiome data to improve diagnostic measures (48).

Currently, treatment modalities include NSAIDs to reduce inflammation, corrective surgery (e.g., arthroplasty, synovectomy, arthroscopy), and routine physical therapy for mobility improvement (45,49). Despite the current treatments, they are temporary, and the disease is long-term.

Autoimmune disease is an umbrella classification for several diseases related to the body’s propensity to misidentify bodily functions, which leads to inappropriately attacking healthy cells in the body. That mistaken attack is a continuous, cyclical overreaction creating pain, swelling, tiredness, mental fatigue, disorientation, and gastrointestinal dysregulation (44). Approximately 3% to 5% of the population has a form of autoimmune disease that may be clinically diagnosed (50). As with rheumatoid arthritis, autoimmune diseases are multifactorial, with genetics, environmental factors, tobacco use, lifestyle choices, socioeconomic factors, contributing to disease onset and negatively impacting the body’s immunological function (47,51). Studies have identified several genetic variants associated with autoimmune disease. Variants in GPR174 (G-protein coupled receptor) and ITM2A (integral membrane protein 2A) (both genes are found on the X chromosome) have been reported in association with autoimmune thyroid diseases in Korean children; DHCR7, GC, CYP2R1 and CYP24A1 affecting the vitamin D pathway (metabolic) leading to type I diabetes predisposition, and interleukin genes, TLR genes, TNF-alpha genes, MYO9B (Myosin IXB expressed in intestinal epithelial cells and is associated with gastrointestinal related autoimmune diseases such as celiac disease and inflammatory bowel disease) (51). These studies highlight the importance of genetic studies in understanding disease pathogenesis and predisposition (51). Although there is no cure for autoimmune disease, there is a hope for remission in rare cases. Examples of autoimmune disease include Lupus, Crohn’s disease, Sjögren’s syndrome, and inflammatory bowel disease.

The human temporomandibular joint (TMJ) is the complex, multifaceted, anatomical craniofacial structure which consists of an articular disc, condyle, and glenoid fossa. It has “three distinct developmental stages: blastematic (weeks 7-8), cavitation (weeks 9-11) and maturation (post-week 12) (52). At approximately 3-months gestation, there is a noticeable mesenchymal condensation forming two regions (blastemata: condylar and temporal). Dorsolateral growth allows for gap closure and the beginning of ossification within the temporal blastema region. The process may be identified by the formation of a cleft superior to the condylar condensate, thus forming the inferior joint cavity. The progression of differentiation forms condylar cartilage and a secondary cleft positioned to the temporal ossification, creating the upper joint cavity and primitive articular disc (53). Due to the differences in developmental stages and embryology, chondrocytes of the primary cartilage undergo interstitial proliferation, whereas secondary cartilage experiences appositional proliferation (54).

As the human TMJ is comprised of an organized network of cartilage, bone, muscle, and synovial fluid, its development is unique in comparison to the body’s other synovial joints of long bones: TMJ has fibrocartilage of both type I and type II collagen covering the articular surface, in contrast, hyaline cartilage containing only type II collagen of the articular surfaces is found within the remaining synovial joints. Tightly woven fibers of the TMJ aid resistance to wear and tear, however, it may be possible that the fibrocartilage composition influences degeneration of the disc (55).

As previously mentioned, the development of the TMJ is complex and it is not confined solely to the developmental processes described above, however, each micro-structure works together to form a multifaceted joint responsible for sliding-hinge mobility (additional terminology regarding mobility are also used to describe a multitude of movements the joint participates in) to aid mastication, allow for oral communication, and breathing, facial expressions, through rotation and translation of the joint. The joint is supported by, and mechanically utilizes, various muscles such as the pterygoid, masseter, and temporalis muscles for structural integrity and function. The region is protected by synovial fluid rich in lubricin; the proteoglycan-4 (PRG-4) based lubricin lubricates the area to reduce friction, serves as a shock absorber, and protects surrounding tissues (56). To further understand the TMJ disc, histological analyses may be performed by utilizing methods such as Hematoxylin and Eosin staining to reveal histopathological degeneration and immunohistochemical staining to detect targeted protein markers and antigens of interest (57).

Temporomandibular Disorder is a multifactorial condition of the temporomandibular joint with an unclear etiology (58). Currently, classification of the signs and symptoms of TMD are heavily reliant upon individuals presenting with localized craniofacial pain caused by, but not limited to hormone differences (controversial and still pending additional analyses), genetics, and/or environmental factors (e.g., trauma, external periodic mal-positioning of ill-fitting devices, or severe malocclusion) (58). Clinical presentations of TMD signs and symptoms include chronic TMJ regional pain, arthritis, disc alterations (e.g., degeneration), anatomical dysfunction or derangement (e.g., malalignment, protrusion, retraction), stress, teeth grinding (bruxism), teeth clenching, popping/clicking/crepitus, trismus (trouble opening or closing the mouth/functional limitation or hinderance of the jaw), auricular dysfunction, and/or trauma causalities (59). TMD’s multifactorial causality can negatively impact a person’s quality of life, yet has non-definitive clinical diagnosis conditions and elusive biomarkers, and it presents a limited availability of non-invasive interventional and preventative measures (55). TMD pathophysiology may be influenced by genetic conditions, as genetic variations/alterations have been detected, later causing physical deterioration and inflammation surrounding the underlying bone (60,61). Studies have explored the impact of genetic polymorphisms involved in TMD and the increased likelihood of diagnosis for women by testing the human estrogen receptor alpha (ERa) gene polymorphisms of *PVULL* and *XBAL* (60). The selected SNPs for evaluation were also reported to be associated with low bone mineral density, osteoarthritis (OA), rheumatoid arthritis (RHA), and TMDs (60). Researchers also found that estrogen increases proinflammatory cytokines and promotes degenerative changes of the TMJ disc in mice, as well as negatively impacting proteoglycans (important for TMJ disk health and salivary function) and articular cartilage, and increasing the incidence of pain associated with TMD due to the increased responsiveness of estrogen (60). The catechol-o-methyltransferase (*COMT*) is an enzyme related to catecholamines that are responsible for neuronal sensory recognition such as pain; reduced activity of COMT is related to an increase of pain recognition due to increased catecholamine, therefore, the COMT gene is of interest for TMD related pain studies and TMD association studies such as our present study (60). Understanding which gene associations are involved in disease onset and/or progression may also lead to improved knowledge about individual predisposition and severity (62).

Periodontal disease is a multifactorial, chronic, and systemic inflammatory condition characterized by the onset of a bacterial invasion, initiating the development of gingivitis (inflammation of the gingiva) (7). As the inflammatory response proceeds, the bacteria thrives in the host environment to continue expansion. Bacterial infiltration has the potential to weaken the immune system to ensure survival (63). Once it increases in severity and extends beyond the gingiva, the disease is then classified as periodontal disease which may also be referred to as periodontitis. Untreated periodontitis can develop into the destruction of the tooth supporting structures (64). Patients left untreated have experienced damaged/destroyed periodontal ligament, tooth roots (cementum), tooth loss, and damage to the surrounding alveolar bone (7). In addition to the effects of bacterial infection and plaque, poor oral hygiene habits, tobacco use, comorbid inflammatory disease, genetics, also contribute to disease (63).

Patients with periodontal disease present clinical symptoms of halitosis (bad breath), difficulty eating, bleeding gingiva, gingiva appear to have a dark-red, wine appearance, and generalized gingival pain/swelling, and eventually tooth loss,. The CDC reports that 47.2% adults 30 years and older in the United States is suffering from periodontitis, and a stark increase in affected individuals is observed as the population ages (65). The report states that 70.1% of adults in the United States 65 years old and above have periodontal disease (65). With regards to the periodontal scoring system, approximately 40% of the population worldwide have mild or moderate periodontal disease, and 19% (greater than 1 billion individuals) have the severe form of the disease (66,67).

The onset of periodontal disease may have a detrimental impact systemically and locally. As an example of the severity of periodontal disease and its potential to influence a systemic outcome, is cardiovascular disease. It is hypothesized that as the periodontal disease develops, the bacteria triggers an inflammatory cascade, traverses to the blood stream, initiates the process of atherosclerosis (arterial blockage accumulation due to the hyper-deposition of plaque; a subset may be referred to ASCVD (atherosclerotic cardiovascular disease), thus leading to cardiovascular disease (CVD) development and progression (68). CVD is a chronic, systemic disease associated with the development of arterial inflammation of the heart and supporting vessels. The development of the disease can lead to destruction of blood supply from the heart to supporting regions within the circulatory pathway, dysfunction of the endothelium, and apoptosis, resulting in death (69). A familial/heritable influence is probable within the disease, as a study evaluating siblings for the presence of periodontal disease revealed a 50% diagnosis within the group; two identical twins within the observed sibling group revealed twin discordance and only one was diagnosed with the disease (64). The influence of genetic factors is evident, as a considerable number of siblings presented with the disease, however, the influence of additional factors are to be considered (e.g., environmental factors) for disease predisposition (64).

In an additional genetics study of approximately fifty-thousand twins, family members, and case controls, approximately 7-38% of the phenotypic variance present was attributable to genetics (64). Numerous genome wide association studies (GWAS) have also unveiled the significance of genetics in the predisposition, onset, and severity of periodontal disease. Polymorphisms in Cyclin dependent KiNase 2B AntiSense RNA (*CDKN2BAS*), Antisense RNA in the Ink Locus (*ANRIL*), glycosyltransferase 6 domain containing 1 (*GLT6D1*), and cyclo-oxygenase 2 (*COX2*) have been found to play a major role in the disease, however, additional studies are needed to substantiate the findings (66). *CDKN2BAS* is responsible for encoding two other genes (*CDKN2A* and *CDKN2B*) that serve as encoders for the protein inhibitors of cyclin-dependent kinase. The role of the gene(s) are essential for apoptosis, cellular proliferation regulation, and extracellular matrix deposition via the TGF-beta signaling pathway (70). Researchers have used *ANRIL* (chromosome 9p21 locus) *as a marker* for association with the development of aggressive periodontal disease; as strong associations were identified in European and African populations (71). *GLT6D1* gene variant has reported in association with mild to aggressive periodontitis; the gene family includes the ABO blood group (72). *COX2* is a gene derivative of cyclo-oxygenase that is present almost exclusively after the onset of inflammation to mediate (via prostaglandins) and regulate disease processes (73). Periodontal disease causes an initial adaptive response that triggers the *COX2* pathway, leading to the synthesis of prostaglandin and aiding the destruction of the extracellular matrix and periodontal tissue (74). As a result, treatments designed to inhibit cyclo-oxygenase 2 (*COX2)* have been designed for use with inflammatory diseases to provide an anti-inflammatory effect (73).

References

1. Owen JA, Punt J, Stranford SA, Jones PP, Kuby J. Kuby immunology. 7th ed. New York: W.H. Freeman; 2013.

2. Dancevic CM, McCulloch DR. Current and emerging therapeutic strategies for preventing inflammation and aggrecanase-mediated cartilage destruction in arthritis. Arthritis Res Ther. 2014;16(5):429.

3. Pahwa R GA, Jialal I. Chronic Inflammation [Internet]. Treasure Island, Florida: StatPearls; 2023 [updated August 7, 2023. Available from: <https://www.ncbi.nlm.nih.gov/books/NBK493173/>.

4. Lux R, Awa W, Walter U. An interdisciplinary analysis of sex and gender in relation to the pathogenesis of bronchial asthma. Respir Med. 2009;103(5):637-49.

5. Kamp IV, Loon JV, Droomers M, Hollander A. Residential Environment and Health: A Review of Methodological and Conceptual Issues. Rev Environ Health. 2021;19(3-4):381-401.

6. Schwan B. Responsibility amid the social determinants of health. Bioethics. 2021;35(1):6-14.

7. Hajishengallis G, Chavakis T. Local and systemic mechanisms linking periodontal disease and inflammatory comorbidities. Nature reviews Immunology. 2021;21(7):426-40.

8. Hamza SA, Asif S, Khurshid Z, Zafar MS, Bokhari SAH. Emerging Role of Epigenetics in Explaining Relationship of Periodontitis and Cardiovascular Diseases. Diseases. 2021;9(3):48.

9. Vieira A, Hilands KM, Braun TW. Saving more teeth-a case for personalized care. J Pers Med. 2015;5(1):30-5.

10. Farup PG, Rootwelt H, Hestad K. APOE - a genetic marker of comorbidity in subjects with morbid obesity. BMC Med Genet. 2020;21(1):146.

11. López-Jiménez JJ, Ortega-Cervantes R, Luna-Záizar H, Fletes-Rayas AL, Beltrán-Miranda CP, Troyo-Sanromán R, et al. Genetic biomarkers related to hemarthrosis, inflammation, and cartilage structure in pediatric patients with hemophilic arthropathy. Mol Genet Genomic Med. 2019;7(11):e979.

12. Tan EK. Genetic marker linking inflammation with sporadic Parkinson's disease. Ann Acad Med Singap. 2011;40(2):111-2.

13. Raman K, Chong M, Akhtar-Danesh GG, D'Mello M, Hasso R, Ross S, et al. Genetic markers of inflammation and their role in cardiovascular disease. Can J Cardiol. 2013;29(1):67-74.

14. Mims JW. Asthma: definitions and pathophysiology. Int Forum Allergy Rhinol. 2015;5 Suppl 1:S2-6.

15. McCracken JL, Tripple JW, Calhoun WJ. Biologic therapy in the management of asthma. Curr Opin Allergy Clin Immunol. 2016;16(4):375-82.

16. Denton E, O'Hehir RE, Hew M. The changing global prevalence of asthma and atopic dermatitis. Allergy. 2023;78(8):2079-80.

17. Miller RL, Grayson MH, Strothman K. Advances in asthma: New understandings of asthma's natural history, risk factors, underlying mechanisms, and clinical management. J Allergy Clin Immunol. 2021;148(6):1430-41.

18. di Palmo E, Cantarelli E, Catelli A, Ricci G, Gallucci M, Miniaci A, et al. The Predictive Role of Biomarkers and Genetics in Childhood Asthma Exacerbations. Int J Mol Sci. 2021;22(9).

19. Ntontsi P, Photiades A, Zervas E, Xanthou G, Samitas K. Genetics and Epigenetics in Asthma. Int J Mol Sci. 2021;22(5).

20. Sharma V, Cowan DC. Obesity, Inflammation, and Severe Asthma: an Update. Curr Allergy Asthma Rep. 2021;21(12):46.

21. Andrea M, Susanna B, Francesca N, Enrico M, Alessandra V. The emerging role of type 2 inflammation in asthma. Expert Rev Clin Immunol. 2021;17(1):63-71.

22. Thomas MS, Parolia A, Kundabala M, Vikram M. Asthma and oral health: a review. Aust Dent J. 2010;55(2):128-33.

23. Fleming MA, Kane WJ, Meneveau MO, Ballantyne CC, Levin DE. Food Insecurity and Obesity in US Adolescents: A Population-Based Analysis. Child Obes. 2021;17(2):110-5.

24. Organization WH. Obesity and Overweight. World Health Organization; 2024.

25. Bray GA, Ryan DH. Evidence-based weight loss interventions: Individualized treatment options to maximize patient outcomes. Diabetes Obes Metab. 2021;23 Suppl 1:50-62.

26. Nathan C. Epidemic inflammation: pondering obesity. Mol Med. 2008;14(7-8):485-92.

27. Singer K, Lumeng CN. The initiation of metabolic inflammation in childhood obesity. J Clin Invest. 2017;127(1):65-73.

28. Wani K, Rahman S, Draz H. Editorial: Dysbiosis, obesity, and inflammation: interrelated phenomena causes or effects of metabolic syndrome? Front Endocrinol (Lausanne). 2023;14:1265314.

29. Trang K, Grant SFA. Genetics and epigenetics in the obesity phenotyping scenario. Rev Endocr Metab Disord. 2023;24(5):775-93.

30. Stelzer G, Rosen N, Plaschkes I, Zimmerman S, Twik M, Fishilevich S, et al. The GeneCards Suite: From Gene Data Mining to Disease Genome Sequence Analyses. Curr Protoc Bioinformatics. 2016;54:1.30.1-1..3.

31. Magliano D, Boyko EJ. IDF diabetes atlas. Tenth edition. ed. Brussels: International Diabetes Federation; 2021.

32. Berbudi A, Rahmadika N, Tjahjadi AI, Ruslami R. Type 2 Diabetes and its Impact on the Immune System. Curr Diabetes Rev. 2020;16(5):442-9.

33. Prevention CfDCa. Hispanic or Latino People and Type 2 Diabetes. Centers for Disease Control and Prevention; 2022.

34. Below JE, Parra EJ. Genome-Wide Studies of Type 2 Diabetes and Lipid Traits in Hispanics. Curr Diab Rep. 2016;16(5):41.

35. Yu XX, Liao MQ, Zeng YF, Gao XP, Liu YH, Sun W, et al. Associations of KCNQ1 Polymorphisms with the Risk of Type 2 Diabetes Mellitus: An Updated Meta-Analysis with Trial Sequential Analysis. J Diabetes Res. 2020;2020:7145139.

36. Han LY, Wu QH, Jiao ML, Hao YH, Liang LB, Gao LJ, et al. Associations between single-nucleotide polymorphisms (+45T>G, +276G>T, -11377C>G, -11391G>A) of adiponectin gene and type 2 diabetes mellitus: a systematic review and meta-analysis. Diabetologia. 2011;54(9):2303-14.

37. Prevention CfDCa. Type 2 Diabetes. Centers for Disease Control and Prevention; 2023.

38. Pawlowicz A, Tymczyna-Borowicz B, Ptasiewicz M. The state of the oral health in patients with type 2 diabetes. Pol Merkur Lekarski. 2020;48(283):27-31.

39. Lontchi-Yimagou E, Sobngwi E, Matsha TE, Kengne AP. Diabetes mellitus and inflammation. Curr Diab Rep. 2013;13(3):435-44.

40. Singer ME, Dorrance KA, Oxenreiter MM, Yan KR, Close KL. The type 2 diabetes 'modern preventable pandemic' and replicable lessons from the COVID-19 crisis. Prev Med Rep. 2022;25(1):101636.

41. King AJ. The use of animal models in diabetes research. British Journal of Pharmacology. 2012;166(3):877-94.

42. Meigs JB. The Genetic Epidemiology of Type 2 Diabetes: Opportunities for Health Translation. Curr Diab Rep. 2019;19(8):62.

43. Kirkwood SC, Hockett RD, Jr. Pharmacogenomic biomarkers. Dis Markers. 2002;18(2):63-71.

44. Radu AF, Bungau SG. Management of Rheumatoid Arthritis: An Overview. Cells. 2021;10(11).

45. Gunasekera WM, Kirwan JR. Rheumatoid arthritis: previously untreated early disease. BMJ Clin Evid. 2016;2016.

46. Dedmon LE. The genetics of rheumatoid arthritis. Rheumatology (Oxford). 2020;59(10):2661-70.

47. Chauhan K, Jandu JS, Brent LH, Al-Dhahir MA. Rheumatoid Arthritis. StatPearls. Treasure Island (FL): StatPearls Publishing

Copyright © 2024, StatPearls Publishing LLC.; 2024.

48. Zhang X, Zhang D, Jia H, Feng Q, Wang D, Liang D, et al. The oral and gut microbiomes are perturbed in rheumatoid arthritis and partly normalized after treatment. Nat Med. 2015;21(8):895-905.

49. Gaffo A, Saag KG, Curtis JR. Treatment of rheumatoid arthritis. Am J Health Syst Pharm. 2006;63(24):2451-65.

50. Wang L, Wang FS, Gershwin ME. Human autoimmune diseases: a comprehensive update. J Intern Med. 2015;278(4):369-95.

51. Wasniewska MG, Bossowski A. Special Issue: Autoimmune Disease Genetics. Genes (Basel). 2021;12(12).

52. Purcell P, Joo BW, Hu JK, Tran PV, Calicchio ML, O'Connell DJ, et al. Temporomandibular Joint Formation Requires Two Distinct Hedgehog-Dependent Steps. Proceedings of the National Academy of Sciences - PNAS. 2009;106(43):18297-302.

53. Nanci A. Ten Cate's oral histology : development, structure, and function. Ninth edition. ed. St. Louis, Missouri: Elsevier; 2018.

54. Mérida Velasco JR, Rodríguez Vázquez JF, De la Cuadra Blanco C, Campos López R, Sánchez M, Mérida Velasco JA. Development of the mandibular condylar cartilage in human specimens of 10–15 weeks’ gestation. Journal of anatomy. 2009;214(1):56-64.

55. Wadhwa S, Kapila S. TMJ disorders: future innovations in diagnostics and therapeutics. J Dent Educ. 2008;72(8):930-47.

56. Xu Y, Zhan J-M, Zheng Y-H, Han Y, Zhang Z-G, Xi C. Computational synovial dynamics of a normal temporomandibular joint during jaw opening. Journal of the Formosan Medical Association. 2013;112(6):346-51.

57. Leonardi R, Musumeci G, Sicurezza E, Loreto C. Lubricin in human temporomandibular joint disc: An immunohistochemical study. Archives of oral biology. 2012;57(6):614-9.

58. List T, Jensen RH. Temporomandibular disorders: Old ideas and new concepts. Cephalalgia. 2017;37(7):692-704.

59. Ibi M. Inflammation and Temporomandibular Joint Derangement. Biol Pharm Bull. 2019;42(4):538-42.

60. Meloto CB, Serrano PO, Ribeiro-DaSilva MC, Rizzatti-Barbosa CM. Genomics and the new perspectives for temporomandibular disorders. Arch Oral Biol. 2011;56(11):1181-91.

61. Li DTS, Leung YY. Temporomandibular Disorders: Current Concepts and Controversies in Diagnosis and Management. Diagnostics (Basel). 2021;11(3).

62. Menezes-Silva R, Khaliq S, Deeley K, Letra A, Vieira AR. Genetic Susceptibility to Periapical Disease: Conditional Contribution of MMP2 and MMP3 Genes to the Development of Periapical Lesions and Healing Response. Journal of endodontics. 2012;38(5):604-7.

63. Janakiram C, Dye BA. A public health approach for prevention of periodontal disease. Periodontol 2000. 2020;84(1):202-14.

64. Vieira A. Genetic Basis of Oral Health Conditions. 1st 2019. ed. Cham: Springer International Publishing AG; 2019.

65. Eke PI DB, Weil L, Thornton-Evans G, Genco R. Prevalence of Periodontitis in Adults in the Uniterd States: 2009 and 2010. Journal of Dental Research. 2012;91(10):914-20.

66. Vaithilingam RD, Safii SH, Baharuddin NA, Ng CC, Cheong SC, Bartold PM, et al. Moving into a new era of periodontal genetic studies: relevance of large case-control samples using severe phenotypes for genome-wide association studies. J Periodontal Res. 2014;49(6):683-95.

67. Haresaku S, Chishaki A, Hatakeyama J, Yoshinaga Y, Yoshizumi J, Yamamoto M, et al. Current status and factors of periodontal disease among Japanese high school students: a cross-sectional study. BDJ Open. 2023;9(1):29.

68. Zardawi F, Gul S, Abdulkareem A, Sha A, Yates J. Association Between Periodontal Disease and Atherosclerotic Cardiovascular Diseases: Revisited. Frontiers in cardiovascular medicine. 2021;7:625579-.

69. Schulz S, Zielske M, Schneider S, Hofmann B, Schaller H-G, Schlitt A, et al. Polymorphism of CD14 Gene Is Associated with Adverse Outcome among Patients Suffering from Cardiovascular Disease. Mediators of inflammation. 2021;2021:3002439-10.

70. Sharif NA. Gene therapies and gene product-based drug candidates for normalizing and preserving tissue functions in animal models of ocular hypertension and glaucoma. Molecular Aspects of Medicine. 2023;94:101218.

71. Linden GJ, Hughes F, Patterson C, Lundy F, Taylor JJ, Preshaw PM, et al. ANRIL Variants and Aggressive Periodontitis in European and African Populations2014.

72. Hashim NT, Linden GJ, Ibrahim ME, Gismalla BG, Lundy FT, Hughes FJ, et al. Replication of the association of GLT6D1 with aggressive periodontitis in a Sudanese population. J Clin Periodontol. 2015;42(4):319-24.

73. Mitchell JA, Warner TD. Cyclo-oxygenase-2: pharmacology, physiology, biochemistry and relevance to NSAID therapy. Br J Pharmacol. 1999;128(6):1121-32.

**Keyword Filters: Temporomandibular Disorder (TMD) Signs and Symptoms**

|  | |
| --- | --- |
| Pop | Discomfort when chewing |
| Click | Clenching |
| Left | Jaw |
| Right | Locks |
| Open | Tiredness from opening |
| Pain | Bruxism |
| Soreness | Tender |
| Excursion and/or translation | 21 |
| Trismus | 21. Yes |
| Late/early/mid | Pressure |
| Opening and closing | Deviation/rolling of one side |
| Shift of the mandible | Dentation on closing |
| Crunching | Snaps |
| 21 (is blank) | Cracking |
| Crepitus | Subluxation |

**Results of Allelic Association Tests in Group 1 (without p-value adjustment).**

| **Chromosome** | **Gene** | **Genetic Variant** | **Base Pair** | **Allele 1/2** | **Allele 1 Frequency (cases)** | **Allele 2 Frequency (controls)** | **P-value** | **Odds Ratio (95% CI)** |
| --- | --- | --- | --- | --- | --- | --- | --- | --- |
| 20 | *MMP9* | rs17577 | 46014472 | A/G | 0.1515 | 0.2905 | **4.12E-05** | 0.436  (0.291-0.6535) |
| 6 | *IL17A* | rs3748067 | 52190541 | T/C | 0.05627 | 0.1203 | 0.003 | 0.4362  (0.2472-0.7695) |
| 2 | *IL1-B* | rs1143634 | 112832813 | A/G | 0.2861 | 0.1776 | 0.006 | 1.855  (1.188-2.899) |
| 17 | *AXIN2* | rs3923087 | 65553143 | T/C | 0.3959 | 0.4934 | 0.03 | 0.6729  (0.4749-0.9536) |
| 12 | *MYOH1* | rs10850110 | 109386921 | A/G | 0.1683 | 0.24 | 0.04 | 0.641  (0.4219-0.9738) |
| 1 | *BRINP3* | rs1342913 | 190151895 | G/A | 0.4331 | 0.3671 | 0.13 | 1.317  (0.9256-1.874) |
| 11 | *WNT11* | rs1533767 | 76194756 | A/G | 0.2232 | 0.1824 | 0.27 | 1.288  (0.8213-2.019) |
| 19 | *TGFB1* | rs2241715 | 41350981 | A/C | 0.364 | 0.3267 | 0.38 | 1.18  (0.8143-1.709) |
| 9 | *CA9* | rs2071676 | 35674056 | A/G | 0.2852 | 0.3092 | 0.55 | 0.8913  (0.6114-1.299) |
| 12 | *AQP5* | rs3736309 | 49964271 | G/A | 0.1055 | 0.09333 | 0.65 | 1.146  (0.6323-2.077) |
| 3 | *GSK3B* | rs9879992 | 119993874 | G/A | 0.2888 | 0.2714 | 0.68 | 1.09  (0.7283-1.631) |
| 5 | *IL4* | rs2070874 | 132674018 | T/C | 0.2448 | 0.2468 | 0.96 | 0.9895  (0.6622-1.478) |

gPLINK Allelic Association Test of group 1 (disease phenotype +, TMD -, and PD-) with without p-value adjustment and the application of a Bonferroni correction alpha threshold of 0.001.

**Results of Allelic Association Tests in Group 2 (without p-value adjustment).**

| **Chromosome** | **Gene** | **Genetic Variant** | **Base Pair** | **Allele 1/2** | **Allele 1 Frequency (cases)** | **Allele 2 Frequency (controls)** | **P-value** | **Odds Ratio (95% CI)** |
| --- | --- | --- | --- | --- | --- | --- | --- | --- |
| 20 | *MMP9* | rs17577 | 46014472 | A/G | 0.1308 | 0.2905 | **9.04E-06** | 0.3676  (0.2338-0.578) |
| 17 | *AXIN2* | rs3923087 | 65553143 | T/C | 0.3168 | 0.4934 | **0.00012** | 0.4761  (0.3253-0.6968) |
| 6 | *IL17A* | rs3748067 | 52190541 | T/C | 0.0628 | 0.1203 | 0.02 | 0.4902  (0.2631-0.9136) |
| 2 | *IL1-B* | rs1143634 | 112832813 | A/G | 0.2649 | 0.1776 | 0.03 | 1.668  (1.037-2.684) |
| 12 | *MYOH1* | rs10850110 | 109386921 | A/G | 0.1761 | 0.24 | 0.09 | 0.6766  (0.4314-1.061) |
| 12 | *AQP5* | rs3736309 | 49964271 | G/A | 0.1435 | 0.09333 | 0.12 | 1.628  (0.8824-3.003) |
| 1 | *BRINP3* | rs1342913 | 190151895 | G/A | 0.4182 | 0.3671 | 0.26 | 1.239  (0.8508-1.806) |
| 5 | *IL4* | rs2070874 | 132674018 | T/C | 0.2116 | 0.2468 | 0.37 | 0.8194  (0.5313-1.264) |
| 11 | *WNT11* | rs1533767 | 76194756 | A/G | 0.2143 | 0.1824 | 0.41 | 1.222  (0.7579-1.971) |
| 19 | *TGFB1* | rs2241715 | 41350981 | A/C | 0.3093 | 0.3267 | 0.69 | 0.923  (0.6201-1.374) |
| 9 | *CA9* | rs2071676 | 35674056 | A/G | 0.3005 | 0.3092 | 0.84 | 0.9595  (0.643-1.432) |
| 3 | *GSK3B* | rs9879992 | 119993874 | G/A | 0.2687 | 0.2714 | 0.95 | 0.9862  (0.6419-1.515) |

gPLINK Allelic Association Test of group 2 (disease phenotype+, TMD+, and PD-) with without p-value adjustment and the application of a Bonferroni correction alpha threshold of 0.001.

**Results of Allelic Association Tests in Group 3 (without p-value adjustment).**

| **Chromosome** | **Gene** | **SNP** | **Base Pair** | **Allele 1/2** | **Allele 1 Frequency (cases)** | **Allele 2 Frequency (controls)** | **P-value** | **Odds Ratio (95% CI)** |
| --- | --- | --- | --- | --- | --- | --- | --- | --- |
| 20 | *MMP9* | rs17577 | 46014472 | A/G | 0.1484 | 0.2905 | **2.66E-05** | 0.4256  (0.2834-0.6393) |
| 17 | *AXIN2* | rs3923087 | 65553143 | T/C | 0.3737 | 0.4934 | 0.006 | 0.6126  (0.4317-0.8693) |
| 2 | *IL1-B* | rs1143634 | 112832813 | A/G | 0.2568 | 0.1776 | 0.04 | 1.6  (1.022-2.504) |
| 12 | *MYOH1* | rs10850110 | 109386921 | A/G | 0.1734 | 0.24 | 0.05 | 0.6641  (0.4376-1.008) |
| 12 | *AQP5* | rs3736309 | 49964271 | G/A | 0.141 | 0.09333 | 0.12 | 1.595  (0.8877-2.865) |
| 6 | *IL17A* | rs3748067 | 52190541 | T/C | 0.08229 | 0.1203 | 0.12 | 0.656  (0.3817-1.127) |
| 11 | *WNT11* | rs1533767 | 76194756 | A/G | 0.2172 | 0.1824 | 0.34 | 1.244  (0.7925-1.952) |
| 1 | *BRINP3* | rs1342913 | 190151895 | G/A | 0.3962 | 0.3671 | 0.49 | 1.131  (0.794-1.611) |
| 9 | *CA9* | rs2071676 | 35674056 | A/G | 0.2879 | 0.3092 | 0.60 | 0.9031  (0.6196-1.316) |
| 5 | *IL4* | rs2070874 | 132674018 | T/C | 0.266 | 0.2468 | 0.62 | 1.106  (0.7421-1.649) |
| 19 | *TGFB1* | rs2241715 | 41350981 | A/C | 0.3096 | 0.3267 | 0.68 | 0.9245  (0.6366-1.343) |
| 3 | *GSK3B* | rs9879992 | 119993874 | G/A | 0.2839 | 0.2714 | 0.76 | 1.064  (0.7107-1.593) |

gPLINK Allelic Association Test of group 3 (disease phenotype+, TMD-, and PD+) with without p-value adjustment and the application of a Bonferroni correction alpha threshold of 0.001.

**Results of Allelic Association Tests in Group 4 (without p-value adjustment).**

| **Chromosome** | **Gene** | **SNP** | **Base Pair** | **Allele 1/2** | **Allele 1 Frequency (cases)** | **Allele 2 Frequency (controls)** | **P-value** | **Odds Ratio (95% CI)** |
| --- | --- | --- | --- | --- | --- | --- | --- | --- |
| 1 | *BRINP3* | rs1554286 | 206770888 | G/A | 0.2545 | 0.5974 | **1.01E-14** | 0.23  (0.1564-0.3383) |
| 17 | *AXIN2* | rs3923087 | 65553143 | T/C | 0.3356 | 0.4934 | **0.00054** | 0.5186  (0.3565-0.7544) |
| 20 | *MMP9* | rs17577 | 46014472 | A/G | 0.1771 | 0.2905 | 0.003 | 0.5256  (0.3419-0.8081) |
| 2 | *IL1-B* | rs1143634 | 112832813 | A/G | 0.2808 | 0.1776 | 0.01 | 1.807  (1.131-2.889) |
| 6 | *IL17A* | rs3748067 | 52190541 | T/C | 0.06277 | 0.1203 | 0.02 | 0.49  (0.2664-0.9011) |
| 12 | *AQP5* | rs3736309 | 49964271 | G/A | 0.1645 | 0.09333 | 0.03 | 1.913  (1.047-3.494) |
| 5 | *IL4* | rs2070874 | 132674018 | T/C | 0.1996 | 0.2468 | 0.22 | 0.761  (0.4932-1.174) |
| 11 | *WNT11* | rs1533767 | 76194756 | A/G | 0.224 | 0.1824 | 0.29 | 1.293  (0.8058-2.076) |
| 12 | *MYOH1* | rs10850110 | 109386921 | A/G | 0.2031 | 0.24 | 0.34 | 0.8072  (0.52-1.253) |
| 9 | *CA9* | rs2071676 | 35674056 | A/G | 0.2804 | 0.3092 | 0.50 | 0.8707  (0.5838-1.298) |
| 3 | *GSK3B* | rs9879992 | 119993874 | G/A | 0.2948 | 0.2714 | 0.59 | 1.122  (0.7348-1.713) |
| 19 | *TGFB1* | rs2241715 | 41350981 | A/C | 0.3297 | 0.3267 | 0.95 | 1.014  (0.6844-1.502) |

gPLINK Allelic Association Test of group 4 (disease phenotype+, TMD+, and PD+) with without p-value adjustment and the application of a Bonferroni correction alpha threshold of 0.001.

**Hardy Weinberg Equilibrium (HWE)** **for Systemic Disease Phenotype+, TMD-, and PD-.**

| **Chromosome** | **Gene** | **Genetic Variant** | **Allele 1/2** | **Genotype** | **Observed**  **Heterozygosity (HET)** | **Expected**  **Heterozygosity (HET)** | **P-value** |
| --- | --- | --- | --- | --- | --- | --- | --- |
| 15 | *ADAM10* | rs653765 | C/T | 48/17/12 | 0.2208 | 0.3907 | **0.0002** |
| 1 | *IL10* | rs1554286 | G/A | 32/28/17 | 0.3636 | 0.481 | **0.03** |
| 16 | *MMP2* | rs243847 | C/T | 9/48/22 | 0.6076 | 0.4865 | **0.04** |
| 20 | *MMP9* | rs17577 | A/G | 9/25/40 | 0.3378 | 0.4123 | 0.15 |
| 2 | *IL1-B* | rs1143634 | A/G | 4/19/53 | 0.25 | 0.2922 | 0.23 |
| 11 | *WNT11* | rs1533767 | A/G | 4/19/51 | 0.2568 | 0.2983 | 0.24 |
| 6 | *IL17A* | rs3748067 | T/C | 2/15/62 | 0.1899 | 0.2116 | 0.30 |
| 12 | *MYOH1* | rs10850110 | A/G | 6/24/45 | 0.32 | 0.3648 | 0.34 |
| 5 | *IL4* | rs2070874 | T/C | 6/26/45 | 0.3377 | 0.3717 | 0.38 |
| 19 | *TGFB1* | rs2241715 | A/C | 9/31/35 | 0.4133 | 0.4399 | 0.60 |
| 1 | *BRINP3* | rs1342913 | G/A | 9/40/30 | 0.5063 | 0.4647 | 0.63 |
| 17 | *AXIN2* | rs3923087 | T/C | 19/37/20 | 0.4868 | 0.4999 | 0.82 |
| 3 | *GSK3B* | rs9879992 | G/A | 5/28/37 | 0.4 | 0.3955 | 1 |
| 9 | *CA9* | rs2071676 | A/G | 7/33/36 | 0.4342 | 0.4272 | 1 |
| 12 | *AQP5* | rs3736309 | G/A | 0/14/61 | 0.1867 | 0.1692 | 1 |

Of the fifteen SNPs tested, three (*IL10* rs1554286, *ADAM10* rs653765, and *MMP2* rs243847) showed deviation of HWE in the comparison group and were excluded from further analyses. Test performed is the unaffected test. CHR=Chromosome

**Hardy-Weinberg Equilibrium for Systemic Disease Phenotype+, TMD+, and PD-.**

| **Chromosome** | **Gene** | **SNP** | **Allele 1/2** | **Genotype** | **Observed**  **Heterozygosity (HET)** | **Expected**  **Heterozygosity (HET)** | **P-value** |
| --- | --- | --- | --- | --- | --- | --- | --- |
| 15 | *ADAM10* | rs653765 | C/T | 48/17/12 | 0.2208 | 0.3907 | **0.0002** |
| 1 | *IL10* | rs1554286 | G/A | 32/28/17 | 0.3636 | 0.481 | **0.03** |
| 16 | *MMP2* | rs243847 | C/T | 9/48/22 | 0.6076 | 0.4865 | **0.04** |
| 20 | *MMP9* | rs17577 | A/G | 9/25/40 | 0.3378 | 0.4123 | 0.16 |
| 2 | *IL1-B* | rs1143634 | A/G | 4/19/53 | 0.25 | 0.2922 | 0.23 |
| 11 | *WNT11* | rs1533767 | A/G | 4/19/51 | 0.2568 | 0.2983 | 0.24 |
| 6 | *IL17A* | rs3748067 | T/C | 2/15/62 | 0.1899 | 0.2116 | 0.30 |
| 12 | *MYOH1* | rs10850110 | A/G | 6/24/45 | 0.32 | 0.3648 | 0.34 |
| 5 | *IL4* | rs2070874 | T/C | 6/26/45 | 0.3377 | 0.3717 | 0.38 |
| 19 | *TGFB1* | rs2241715 | A/C | 9/31/35 | 0.4133 | 0.4399 | 0.60 |
| 1 | *BRINP3* | rs1342913 | G/A | 9/40/30 | 0.5063 | 0.4647 | 0.63 |
| 17 | *AXIN2* | rs3923087 | T/C | 19/37/20 | 0.4868 | 0.4999 | 0.82 |
| 3 | *GSK3B* | rs9879992 | G/A | 5/28/37 | 0.4 | 0.3955 | 1 |
| 9 | *CA9* | rs2071676 | A/G | 7/33/36 | 0.4342 | 0.4272 | 1 |
| 12 | *AQP5* | rs3736309 | G/A | 0/14/61 | 0.1867 | 0.1692 | 1 |

*Test performed is the unaffected test.

**Hardy-Weinberg Equilibrium for Systemic Disease Phenotype+, TMD-, and PD+.**

| **Chromosome** | **Gene** | **Genetic Variant** | **Allele 1/2** | **Genotype** | **Observed**  **Heterozygosity (HET)** | **Expected**  **Heterozygosity (HET)** | **P-value** |
| --- | --- | --- | --- | --- | --- | --- | --- |
| 15 | *ADAM10* | rs653765 | C/T | 48/17/12 | 0.2208 | 0.3907 | **0.00024** |
| 1 | *IL10* | rs1554286 | G/A | 32/28/17 | 0.3636 | 0.481 | **0.03413** |
| 16 | *MMP2* | rs243847 | C/T | 9/48/22 | 0.6076 | 0.4865 | **0.03814** |
| 20 | *MMP9* | rs17577 | A/G | 9/25/40 | 0.3378 | 0.4123 | 0.16 |
| 2 | *IL1-B* | rs1143634 | A/G | 4/19/53 | 0.25 | 0.2922 | 0.23 |
| 11 | *WNT11* | rs1533767 | A/G | 4/19/51 | 0.2568 | 0.2983 | 0.24 |
| 6 | *IL17A* | rs3748067 | T/C | 2/15/62 | 0.1899 | 0.2116 | 0.30 |
| 12 | *MYOH1* | rs10850110 | A/G | 6/24/45 | 0.32 | 0.3648 | 0.34 |
| 5 | *IL4* | rs2070874 | T/C | 6/26/45 | 0.3377 | 0.3717 | 0.38 |
| 19 | *TGFB1* | rs2241715 | A/C | 9/31/35 | 0.4133 | 0.4399 | 0.60 |
| 1 | *BRINP3* | rs1342913 | G/A | 9/40/30 | 0.5063 | 0.4647 | 0.63 |
| 17 | *AXIN2* | rs3923087 | T/C | 19/37/20 | 0.4868 | 0.4999 | 0.82 |
| 9 | *CA9* | rs2071676 | A/G | 7/33/36 | 0.4342 | 0.4272 | 1 |
| 12 | *AQP5* | rs3736309 | G/A | 0/14/61 | 0.1867 | 0.1692 | 1 |
| 3 | *GSK3B* | rs9879992 | G/A | 5/28/37 | 0.4 | 0.3955 | 1 |

*Test performed is the unaffected test.

**Hardy-Weinberg Equilibrium for Systemic Disease Phenotype+, TMD+, and PD+.**

| **Chromosome** | **Gene** | **Genetic Variant** | **Allele 1/2** | **Genotype** | **Observed**  **Heterozygosity (HET)** | **Expected**  **Heterozygosity (HET)** | **P-value** |
| --- | --- | --- | --- | --- | --- | --- | --- |
| 15 | *ADAM10* | rs653765 | C/T | 48/17/12 | 0.2208 | 0.3907 | **0.0002** |
| 1 | *IL10* | rs1554286 | G/A | 32/28/17 | 0.3636 | 0.481 | **0.03** |
| 16 | *MMP2* | rs243847 | C/T | 9/48/22 | 0.6076 | 0.4865 | **0.04** |
| 20 | *MMP9* | rs17577 | A/G | 9/25/40 | 0.3378 | 0.4123 | 0.16 |
| 2 | *IL1-B* | rs1143634 | A/G | 4/19/53 | 0.25 | 0.2922 | 0.23 |
| 11 | *WNT11* | rs1533767 | A/G | 4/19/51 | 0.2568 | 0.2983 | 0.24 |
| 6 | *IL17A* | rs3748067 | T/C | 2/15/62 | 0.1899 | 0.2116 | 0.30 |
| 12 | *MYOH1* | rs10850110 | A/G | 6/24/45 | 0.32 | 0.3648 | 0.34 |
| 5 | *IL4* | rs2070874 | T/C | 6/26/45 | 0.3377 | 0.3717 | 0.38 |
| 19 | *TGFB1* | rs2241715 | A/C | 9/31/35 | 0.4133 | 0.4399 | 0.60 |
| 1 | *BRINP3* | rs1342913 | G/A | 9/40/30 | 0.5063 | 0.4647 | 0.63 |
| 17 | *AXIN2* | rs3923087 | T/C | 19/37/20 | 0.4868 | 0.4999 | 0.82 |
| 3 | *GSK3B* | rs9879992 | G/A | 5/28/37 | 0.4 | 0.3955 | 1 |
| 9 | *CA9* | rs2071676 | A/G | 7/33/36 | 0.4342 | 0.4272 | 1 |
| 12 | *AQP5* | rs3736309 | G/A | 0/14/61 | 0.1867 | 0.1692 | 1 |

* Test performed is the unaffected test.

**DNA Extraction from Whole Saliva**

**Day 1**

1. Bring samples up to room temperature.
2. Centrifuge for 5 minutes at 10,000xg at room temperature to pull out the buccal cells.
3. Discard the supernatant and add 1ml of extraction buffer (Tris-HCL 10mM, pH7.8; EDTA 5mM; SDS 0.5%)
4. Vortex samples (at this point, can be frozen for later use)
5. Add 5ul of proteinase K (20mg/ml), agitate by hand shaking
6. Incubate over night at 56°C

**Day 2**

1. Vortex after removing from water bath.
2. Carefully add 500ul of 10M Ammonium Acetate to the side of the tube to remove undigested proteins, mix by inverting the tube 3-5 minutes.
3. Centrifuge for 15 minutes at 21,000xg.
4. Transfer the supernatant to two Eppendorf’s.
5. Add a volume of cold isopropanol equal to the volume of supernatant (typically 700ul) and shake vigorously.
6. Incubate at -20°C for 30 minutes. [This is the minimum amount of time they need to be at -20°C, they can be left here for days if necessary]
7. Centrifuge at 10,000xg rpm for 20 minutes at 4°C
8. Pour off the supernatant, wash with 1ml cold 70% ethanol (on ice) and shake vigorously.
9. Centrifuge at 10,000xg for 5 minutes at 4°C
10. Pour off supernatant, allow to air dry for 45 to 60 minutes.
11. Re-suspend in 100ul TE buffer, vortex to dissolve pellet. [If the pellet is not dissolving, incubate at 56°C for 15 minutes.]

| **Centrifuge name** | **10,000 x g** | **21,000 x g** |
| --- | --- | --- |
| 5810R | 7,049 rpm | XXXXX |
| 5417R | 9,703 rpm | 14,061 rpm |
| 5424 | 10,319 rpm | 14,954 rpm |

Extraction Buffer Solution:

|  | **1 L** | **500 ml** |
| --- | --- | --- |
| Tris-HCl | 10ml of 1M | 5ml |
| EDTA | 1.86g | 0.93g |
| SDS | 5g | 2.5g |

***Source****: Garbieri TF, Brozoski DT, Dionísio TJ, Santos CF, Neves LT. Human DNA extraction from whole saliva that was fresh or stored for 3, 6 or 12 months using five different protocols. J Appl Oral Sci. 2017 Mar-Apr;25(2):147-158. doi: 10.1590/1678-77572016-0046.*

**Real Time Allelic Discrimination Genotyping**

Setting up the Reaction

1. Dilute DNA samples to 2ng/µl in a 96 well plate.
2. Make a Reaction Mix based on the following formula, allowing at least 4 extra wells for a negative control and general loss:

Master Mix – 1.5µl

40X SNP - 0.037 µl

Water - 0.462µl

total - 2 µl per well

*Note: if using multiple SNPs, make multiple mixes. Do not put more than one SNP in a mix and do not put more than one mix in a well*

1. Add 1µl of diluted DNA from the 96 well plate to the 384 well plate with the multi-channel pipettor
2. Add 2µl of the reaction mix to each well with the electronic repeater pipettor.
3. Make sure one well has the 2µl of mix but no DNA. Add 1µl of water instead. This will be your negative control.
4. Remove adhesive back from the clear optical film and press it onto the plate using the plastic spatula. **Do not rub it onto the plate with your fingers.** Make sure the film is centered on the plate, if it is too close to anyone side you run the risk of sample evaporation. The film must be designated “optical or the laser will not be able to penetrate.
5. Run the plate in the thermocycler on the “assay1program:

95ºC for 10 minutes, 40 Cycles of [92ºC for 15 seconds and 60ºC for 1 minute]

**Bibliography**

74. Lazăr L, Loghin A, Bud ES, Cerghizan D, Horváth E, Nagy EE. Cyclooxygenase-2 and matrix metalloproteinase-9 expressions correlate with tissue inflammation degree in periodontal disease. Rom J Morphol Embryol. 2015;56(4):1441-6.

75. Gasmi Benahmed A, Gasmi A, Tippairote T, Mujawdiya PK, Avdeev O, Shanaida Y, et al. Metabolic Conditions and Peri-Implantitis. Antibiotics (Basel). 2022;12(1).

76. Casado PL, Aguiar DP, Costa LC, Fonseca MA, Vieira TC, Alvim-Pereira CC, et al. Different contribution of BRINP3 gene in chronic periodontitis and peri-implantitis: a cross-sectional study. BMC Oral Health. 2015;15:33.

77. Lee J, Keam B, Jang EJ, Park MS, Lee JY, Kim DB, et al. Development of a predictive model for type 2 diabetes mellitus using genetic and clinical data. Osong Public Health Res Perspect. 2011;2(2):75-82.

78. Galimova E, Rätsep R, Traks T, Kingo K, Escott-Price V, Kõks S. Interleukin-10 family cytokines pathway: genetic variants and psoriasis. Br J Dermatol. 2017;176(6):1577-87.

79. Krishna Priya EK, Srinivas L, Rajesh S, Sasikala K, Banerjee M. Pro-inflammatory cytokine response pre-dominates immuno-genetic pathway in development of rheumatoid arthritis. Mol Biol Rep. 2020;47(11):8669-77.

80. da Silva FRP, Vasconcelos A, de Carvalho França LF, Di Lenardo D, Nascimento HMS, Vasconcelos DFP. Association between the rs1143634 polymorphism in interleukin-1B and chronic periodontitis: Results from a meta-analysis composed by 54 case/control studies. Gene. 2018;668:97-106.

81. Andrade Filho PA, Letra A, Cramer A, Prasad JL, Garlet GP, Vieira AR, et al. Insights from studies with oral cleft genes suggest associations between WNT-pathway genes and risk of oral cancer. J Dent Res. 2011;90(6):740-6.

82. Zhu N, Gong Y, Chen XD, Zhang J, Long F, He J, et al. Association between the polymorphisms of interleukin-4, the interleukin-4 receptor gene and asthma. Chin Med J (Engl). 2013;126(15):2943-51.

83. Eskandari-Nasab E, Moghadampour M, Tahmasebi A. Meta-Analysis of Risk Association Between Interleukin-17A and F Gene Polymorphisms and Inflammatory Diseases. J Interferon Cytokine Res. 2017;37(4):165-74.

84. Kuwabara T, Ishikawa F, Kondo M, Kakiuchi T. The Role of IL-17 and Related Cytokines in Inflammatory Autoimmune Diseases. Mediators Inflamm. 2017;2017:3908061.

85. Chien MH, Yang JS, Chu YH, Lin CH, Wei LH, Yang SF, et al. Impacts of CA9 gene polymorphisms and environmental factors on oral-cancer susceptibility and clinicopathologic characteristics in Taiwan. PLoS One. 2012;7(12):e51051.

86. de Freitas EM, Machado RA, de Moura Santos E, de Matos FR, Galvão HC, Miranda Soares PB, et al. Polymorphisms associated with oral clefts as potential susceptibility markers for oral and breast cancer. Arch Oral Biol. 2019;99:9-14.

87. Küchler EC, Reis CLB, Carelli J, Scariot R, Nelson-Filho P, Coletta RD, et al. Potential interactions among single nucleotide polymorphisms in bone- and cartilage-related genes in skeletal malocclusions. Orthod Craniofac Res. 2021;24(2):277-87.

88. Tassopoulou-Fishell M, Deeley K, Harvey EM, Sciote J, Vieira AR. Genetic variation in myosin 1H contributes to mandibular prognathism. Am J Orthod Dentofacial Orthop. 2012;141(1):51-9.

89. Cruz CV, Mattos CT, Maia JC, Granjeiro JM, Reis MF, Mucha JN, et al. Genetic polymorphisms underlying the skeletal Class III phenotype. Am J Orthod Dentofacial Orthop. 2017;151(4):700-7.

90. Sahu A, Swaroop S, Kant S, Banerjee M. Signatures for chronic obstructive pulmonary disease (COPD) and asthma: a comparative genetic analysis. Br J Biomed Sci. 2021;78(4):177-83.

91. Chen YT, Lin CW, Chou YE, Su SC, Chang LC, Lee CY, et al. Potential impact of ADAM-10 genetic variants with the clinical features of oral squamous cell carcinoma. J Cell Mol Med. 2023;27(8):1144-52.

92. Chisini LA, Santos FDC, de Carvalho RV, Horta BL, Tovo-Rodrigues L, Demarco FF, et al. Impact of tooth mineral tissues genes on dental caries: A birth-cohort study. J Dent. 2023;133:104505.

93. Peng S, Yan Y, Li R, Dai H, Xu J. Extracellular vesicles from M1-polarized macrophages promote inflammation in the temporomandibular joint via miR-1246 activation of the Wnt/β-catenin pathway. Ann N Y Acad Sci. 2021;1503(1):48-59.

94. Li X, Li Y, Liu G, Wu W. New insights of the correlation between AXIN2 polymorphism and cancer risk and susceptibility: evidence from 72 studies. BMC Cancer. 2021;21(1):353.

95. Akram M, Sabar MF, Bano I, Ghani MU, Shahid M. Single Nucleotide Polymorphisms Of Transforming Growth Factor-Β1 Gene As Potential Asthma Susceptible Variants In Punjabi Population Of Pakistan. J Ayub Med Coll Abbottabad. 2022;34(Suppl 1)(4):S944-s8.

96. Tziastoudi M, Stefanidis I, Hadjigeorgiou GM, Stravodimos K, Zintzaras E. A systematic review and meta-analysis of genetic association studies for the role of inflammation and the immune system in diabetic nephropathy. Clin Kidney J. 2017;10(3):293-300.

97. Jiménez-Morales S, Martínez-Aguilar N, Gamboa-Becerra R, Jiménez-Ruíz JL, López-Ley D, Lou H, et al. Polymorphisms in metalloproteinase-9 are associated with the risk for asthma in Mexican pediatric patients. Hum Immunol. 2013;74(8):998-1002.

98. Zou F, Zhang J, Xiang G, Jiao H, Gao H. Association of Matrix Metalloproteinase 9 (MMP-9) Polymorphisms with Asthma Risk: A Meta-Analysis. Can Respir J. 2019;2019:9260495.

99. Sethuraman A, Gonzalez NM, Grenier CE, Kansagra KS, Mey KK, Nunez-Zavala SB, et al. Continued misuse of multiple testing correction methods in population genetics-A wake-up call? Mol Ecol Resour. 2019;19(1):23-6.

100. Nikita A, Nikita A, Andrew B, Andrew B, May T, May T. Hardy-Weinberg Equilibrium in the Large Scale Genomic Sequencing Era. Frontiers in genetics. 2020;11.

101. Purcell S, Neale B, Todd-Brown K, Thomas L, Ferreira MA, Bender D, et al. PLINK: a tool set for whole-genome association and population-based linkage analyses. Am J Hum Genet. 2007;81(3):559-75.

102. Zubair N, Conomos MP, Hood L, Omenn GS, Price ND, Spring BJ, et al. Genetic Predisposition Impacts Clinical Changes in a Lifestyle Coaching Program. Sci Rep. 2019;9(1):6805.

103. Liu CC, Ahearn JM. The search for lupus biomarkers. Best Pract Res Clin Rheumatol. 2009;23(4):507-23.

104. Grzela K, Litwiniuk M, Zagorska W, Grzela T. Airway Remodeling in Chronic Obstructive Pulmonary Disease and Asthma: the Role of Matrix Metalloproteinase-9. Arch Immunol Ther Exp (Warsz). 2016;64(1):47-55.

105. Huang H. Matrix Metalloproteinase-9 (MMP-9) as a Cancer Biomarker and MMP-9 Biosensors: Recent Advances. Sensors (Basel). 2018;18(10).

106. Suchankova P, Pettersson R, Nordenström K, Holm G, Ekman A. Personality traits and the R668Q polymorphism located in the MMP-9 gene. Behavioural Brain Research. 2012;228(1):232-5.

107. Nanni S, Melandri G, Hanemaaijer R, Cervi V, Tomasi L, Altimari A, et al. Matrix metalloproteinases in premature coronary atherosclerosis: influence of inhibitors, inflammation, and genetic polymorphisms. Transl Res. 2007;149(3):137-44.

108. Lammi L, Arte S, Somer M, Jarvinen H, Lahermo P, Thesleff I, et al. Mutations in AXIN2 cause familial tooth agenesis and predispose to colorectal cancer. Am J Hum Genet. 2004;74(5):1043-50.

109. Mazzoni SM, Fearon ER. AXIN1 and AXIN2 variants in gastrointestinal cancers. Cancer Lett. 2014;355(1):1-8.

110. da Silva AM, Falcão MML, Freitas VS, Vieira AR. Genetic and environmental contributions for the relationship between tooth loss and oral potentially malignant disorders and oral squamous cell carcinoma. Head Neck. 2024.

111. National Academies of Sciences E, Medicine. Temporomandibular Disorders: Priorities for Research and Care. Bond EC, Mackey S, English R, Liverman CT, Yost O, editors. Washington, DC: The National Academies Press; 2020. 426 p.

112. USA D. Pittsburgh, PA [Website]. Deloitte, Datawheel, Cesar Hidalgo; 2021 [Comprehensive website and visualization engine of public US government data.]. Available from: <https://datausa.io/profile/geo/pittsburgh-pa/>.

113. C.W. Duarte LKV, T. Mark Beasley, H.K. Tiwari,. Multifactorial Inheritance and Complex Diseases,. In: Sciences RMiB, editor. Science Direct Biomedical Sciences,: Elsevier,; 2014.

114. Arcaya MC, Arcaya AL, Subramanian SV. Inequalities in health: definitions, concepts, and theories. Glob Health Action. 2015;8:27106.

115. Haskell WL. Physical activity by self-report: a brief history and future issues. J Phys Act Health. 2012;9 Suppl 1:S5-10.

116. Dong G, Qu L, Gong X, Pang B, Yan W, Wei J. Effect of Social Factors and the Natural Environment on the Etiology and Pathogenesis of Diabetes Mellitus. Int J Endocrinol. 2019;2019:8749291.

117. Emma R, Caruso M, Campagna D, Pulvirenti R, Li Volti G. The Impact of Tobacco Cigarettes, Vaping Products and Tobacco Heating Products on Oxidative Stress. Antioxidants (Basel). 2022;11(9).

Summary of Associations Studies

**Group 1: Disease phenotype +, TMD -, and PD-**

gPLINK Genotypic Association Test of group 1 with the application of a Bonferroni correction alpha threshold of 0.001 to provide the results of genotypic analysis of study participants presenting with only 1 of the 4 disease phenotypes (asthma, rheumatoid arthritis/autoimmune disease, obesity, or type II diabetes) **who are also negative** for both TMD and periodontitis (PD).

**Results of genotypic and allelic association tests in Group 1****.**

| **CHR** | **Gene** | **SNP** | **Allele 1/2** | **Test** | **Affected** | **Unaffected** | **X^2^** | **DF** | **P-value** |
| --- | --- | --- | --- | --- | --- | --- | --- | --- | --- |
| 12 | *AQP5* | rs3736309 | G/A | Allelic | 84/712 | 14/136 | 0.2021 | 1 | 0.65 |
| 12 | *AQP5* | rs3736309 | G/A | Geno | 7/70/321 | 0/14/61 | 1.364 | 2 | 0.51 |
| 12 | *AQP5* | rs3736309 | G/A | Dom | 77/321 | 14/61 | 0.019 | 1 | 0.89 |
| 12 | *AQP5* | rs3736309 | G/A | Rec | 7/391 | 0/75 | 1.339 | 1 | 0.25 |
| 17 | *AXIN2* | rs3923087 | T/C | Dom | 227/167 | 56/20 | 6.868 | 1 | 0.009 |
| 17 | *AXIN2* | rs3923087 | T/C | Allelic | 312/476 | 75/77 | 4.999 | 1 | 0.03 |
| 17 | *AXIN2* | rs3923087 | T/C | Geno | 85/142/167 | 19/37/20 | 7.147 | 2 | 0.03 |
| 17 | *AXIN2* | rs3923087 | T/C | Rec | 85/309 | 19/57 | 0.4341 | 1 | 0.51 |
| 1 | *BRINP3* | rs1342913 | G/A | Rec | 84/312 | 9/70 | 4.033 | 1 | 0.05 |
| 1 | *BRINP3* | rs1342913 | G/A | Allelic | 343/449 | 58/100 | 2.352 | 1 | 0.13 |
| 1 | *BRINP3* | rs1342913 | G/A | Geno | 84/175/137 | 9/40/30 | 4.061 | 2 | 0.13 |
| 1 | *BRINP3* | rs1342913 | G/A | Dom | 259/137 | 49/30 | 0.3298 | 1 | 0.57 |
| 9 | *CA9* | rs2071676 | A/G | Dom | 190/208 | 40/36 | 0.6116 | 1 | 0.43 |
| 9 | *CA9* | rs2071676 | A/G | Allelic | 227/569 | 47/105 | 0.3588 | 1 | 0.55 |
| 9 | *CA9* | rs2071676 | A/G | Geno | 37/153/208 | 7/33/36 | 0.7004 | 2 | 0.70 |
| 9 | *CA9* | rs2071676 | A/G | Rec | 37/361 | 7/69 | 0.0005599 | 1 | 0.98 |
| 3 | *GSK3B* | rs9879992 | G/A | Rec | 37/356 | 5/65 | 0.3718 | 1 | 0.54 |
| 3 | *GSK3B* | rs9879992 | G/A | Allelic | 227/559 | 38/102 | 0.1756 | 1 | 0.68 |
| 3 | *GSK3B* | rs9879992 | G/A | Geno | 37/153/203 | 5/28/37 | 0.372 | 2 | 0.83 |
| 3 | *GSK3B* | rs9879992 | G/A | Dom | 190/203 | 33/37 | 0.03445 | 1 | 0.85 |
| 2 | *IL1-B* | rs1143634 | A/G | Allelic | 206/514 | 27/125 | 7.543 | 1 | 0.006 |
| 2 | *IL1-B* | rs1143634 | A/G | Geno | 52/102/206 | 4/19/53 | 6.024 | 2 | 0.05 |
| 2 | *IL1-B* | rs1143634 | A/G | Dom | 154/206 | 23/53 | 4.075 | 1 | 0.04 |
| 2 | *IL1-B* | rs1143634 | A/G | Rec | 52/308 | 4/72 | 4.725 | 1 | 0.03 |
| 6 | *IL17A* | rs3748067 | T/C | Allelic | 44/738 | 19/139 | 8.607 | 1 | 0.003 |
| 6 | *IL17A* | rs3748067 | T/C | Geno | 1/42/348 | 2/15/62 | 9.852 | 2 | 0.007 |
| 6 | *IL17A* | rs3748067 | T/C | Dom | 43/348 | 17/62 | 6.533 | 1 | 0.01 |
| 6 | *IL17A* | rs3748067 | T/C | Rec | 1/390 | 2/77 | 5.367 | 1 | 0.02 |
| 5 | *IL4* | rs2070874 | T/C | Rec | 26/358 | 6/71 | 0.1036 | 1 | 0.75 |
| 5 | *IL4* | rs2070874 | T/C | Dom | 162/222 | 32/45 | 0.01041 | 1 | 0.92 |
| 5 | *IL4* | rs2070874 | T/C | Geno | 26/136/222 | 6/26/45 | 0.1505 | 2 | 0.93 |
| 5 | *IL4* | rs2070874 | T/C | Allelic | 188/580 | 38/116 | 0.002668 | 1 | 0.96 |
| 20 | *MMP9* | rs17577 | A/G | Geno | 9/102/285 | 9/25/40 | 20.3 | 2 | **0.00004** |
| 20 | *MMP9* | rs17577 | A/G | Allelic | 120/672 | 43/105 | 16.81 | 1 | **0.00004** |
| 20 | *MMP9* | rs17577 | A/G | Rec | 9/387 | 9/65 | 16.56 | 1 | **0.00005** |
| 20 | *MMP9* | rs17577 | A/G | Dom | 111/285 | 34/40 | 9.381 | 1 | 0.002 |
| 12 | *MYOH1* | rs10850110 | A/G | Allelic | 134/662 | 36/114 | 4.397 | 1 | 0.04 |
| 12 | *MYOH1* | rs10850110 | A/G | Dom | 117/281 | 30/45 | 3.312 | 1 | 0.07 |
| 12 | *MYOH1* | rs10850110 | A/G | Geno | 17/100/281 | 6/24/45 | 3.971 | 2 | 0.14 |
| 12 | *MYOH1* | rs10850110 | A/G | Rec | 17/381 | 6/69 | 1.897 | 1 | 0.17 |
| 19 | *TGFB1* | rs2241715 | A/C | Dom | 236/161 | 40/35 | 0.9707 | 1 | 0.32 |
| 19 | *TGFB1* | rs2241715 | A/C | Allelic | 289/505 | 49/101 | 0.7642 | 1 | 0.38 |
| 19 | *TGFB1* | rs2241715 | A/C | Geno | 53/183/161 | 9/31/35 | 0.9707 | 2 | 0.62 |
| 19 | *TGFB1* | rs2241715 | A/C | Rec | 53/344 | 9/66 | 0.1008 | 1 | 0.75 |
| 11 | *WNT11* | rs1533767 | A/G | Allelic | 175/609 | 27/121 | 1.22 | 1 | 0.27 |
| 11 | *WNT11* | rs1533767 | A/G | Geno | 26/123/243 | 4/19/51 | 1.284 | 2 | 0.53 |
| 11 | *WNT11* | rs1533767 | A/G | Dom | 149/243 | 23/51 | 1.283 | 1 | 0.26 |
| 11 | *WNT11* | rs1533767 | A/G | Rec | 26/366 | 4/70 | 0.156 | 1 | 0.69 |

*P-values below 0.001 indicate significant associations (shown in bold). CHR=Chromosome, DF=Degrees of Freedom;Test: Allelic, Geno (Genotypic), Dom (Dominant), and Rec (Recessive).

**Results of allelic association tests in Group 1 (adjusted p-values).**

| **CHR** | **Gene** | **SNP** | **Base Pair** | **Allele 1/2** | **Allele 1**  **Frequency (cases)** | **Allele 2 Frequency (controls)** | **P-value** | **Odds Ratio (95% CI)** |
| --- | --- | --- | --- | --- | --- | --- | --- | --- |
| 20 | *MMP9* | rs17577 | 46014472 | A/G | 0.1515 | 0.2905 | **0.00004** | 0.436  (0.291-0.6535) |
| 6 | *IL17A* | rs3748067 | 52190541 | T/C | 0.05627 | 0.1203 | 0.003 | 0.4362  (0.2472-0.7695) |
| 2 | *IL1-B* | rs1143634 | 112832813 | A/G | 0.2861 | 0.1776 | 0.006 | 1.855  (1.188-2.899) |
| 17 | *AXIN2* | rs3923087 | 65553143 | T/C | 0.3959 | 0.4934 | 0.03 | 0.6729  (0.4749-0.9536) |
| 12 | *MYOH1* | rs10850110 | 109386921 | A/G | 0.1683 | 0.24 | 0.04 | 0.641  (0.4219-0.9738) |
| 1 | *BRINP3* | rs1342913 | 190151895 | G/A | 0.4331 | 0.3671 | 0.13 | 1.317  (0.9256-1.874) |
| 11 | *WNT11* | rs1533767 | 76194756 | A/G | 0.2232 | 0.1824 | 0.27 | 1.288  (0.8213-2.019) |
| 19 | *TGFB1* | rs2241715 | 41350981 | A/C | 0.364 | 0.3267 | 0.38 | 1.18  (0.8143-1.709) |
| 9 | *CA9* | rs2071676 | 35674056 | A/G | 0.2852 | 0.3092 | 0.55 | 0.8913  (0.6114-1.299) |
| 12 | *AQP5* | rs3736309 | 49964271 | G/A | 0.1055 | 0.09333 | 0.65 | 1.146  (0.6323-2.077) |
| 3 | *GSK3B* | rs9879992 | 119993874 | G/A | 0.2888 | 0.2714 | 0.68 | 1.09  (0.7283-1.631) |
| 5 | *IL4* | rs2070874 | 132674018 | T/C | 0.2448 | 0.2468 | 0.96 | 0.9895  (0.6622-1.478) |

*CHR=Chromosome; gPLINK Allelic Association Test of group 1 (disease phenotype +, TMD -, and PD-) with an adjusted p-value and the application of a Bonferroni correction alpha threshold of 0.001.

**Group 2: Disease phenotype+, TMD+, and PD-**

gPLINK Genotypic Association Test of group 2 with the application of a Bonferroni correction alpha threshold of 0.001 to provide the results of genotypic analysis for study participants presenting with only 1 of the 4 disease phenotypes (asthma, rheumatoid arthritis/autoimmune disease, obesity, or type II diabetes), are positive for TMD, and **who are negative** for periodontitis (PD).

**Results of genotypic and allelic association tests in Group 2.**

| **CHR** | **Gene** | **SNP** | **Allele 1/2** | **Test** | **Affected** | **Unaffected** | **X^2^** | **DF** | **P-value** |
| --- | --- | --- | --- | --- | --- | --- | --- | --- | --- |
| 12 | *AQP5* | rs3736309 | G/A | Allelic | 62/370 | 14/136 | 2.47 | 1 | 0.12 |
| 12 | *AQP5* | rs3736309 | G/A | Geno | 9/44/163 | 0/14/61 | 3.455 | 2 | 0.18 |
| 12 | *AQP5* | rs3736309 | G/A | Dom | 53/163 | 14/61 | 1.082 | **1** | 0.30 |
| 12 | *AQP5* | rs3736309 | G/A | Rec | 9/207 | 0/75 | 3.225 | **1** | 0.07 |
| 17 | *AXIN2* | rs3923087 | T/C | Allelic | 128/276 | 75/77 | 14.86 | 1 | **0.0001** |
| 17 | *AXIN2* | rs3923087 | T/C | Dom | 99/103 | 56/20 | 13.63 | 1 | **0.0002** |
| 17 | *AXIN2* | rs3923087 | T/C | Geno | 29/70/103 | 19/37/20 | 14.05 | 2 | **0.0009** |
| 17 | *AXIN2* | rs3923087 | T/C | Rec | 29/173 | 19/57 | 4.379 | 1 | 0.04 |
| 1 | *BRINP3* | rs1342913 | G/A | Rec | 45/169 | 9/70 | 3.564 | 1 | 0.06 |
| 1 | *BRINP3* | rs1342913 | G/A | Geno | 45/89/80 | 9/40/30 | 3.984 | 2 | 0.14 |
| 1 | *BRINP3* | rs1342913 | G/A | Allelic | 179/249 | 58/100 | 1.253 | 1 | 0.26 |
| 1 | *BRINP3* | rs1342913 | G/A | Dom | 134/80 | 49/30 | 0.00861 | 1 | 0.93 |
| 9 | *CA9* | rs2071676 | A/G | Dom | 107/111 | 40/36 | 0.2839 | 1 | 0.59 |
| 9 | *CA9* | rs2071676 | A/G | Rec | 24/194 | 7/69 | 0.1933 | 1 | 0.66 |
| 9 | *CA9* | rs2071676 | A/G | Geno | 24/83/111 | 7/33/36 | 0.7233 | 2 | 0.70 |
| 9 | *CA9* | rs2071676 | A/G | Allelic | 131/305 | 47/105 | 0.0409 | 1 | 0.84 |
| 3 | *GSK3B* | rs9879992 | G/A | Rec | 18/196 | 5/65 | 0.114 | 1 | 0.74 |
| 3 | *GSK3B* | rs9879992 | G/A | Dom | 97/117 | 33/37 | 0.07006 | 1 | 0.79 |
| 3 | *GSK3B* | rs9879992 | G/A | Geno | 18/79/117 | 5/28/37 | 0.27 | 2 | 0.87 |
| 3 | *GSK3B* | rs9879992 | G/A | Allelic | 115/313 | 38/102 | 0.004015 | 1 | 0.95 |
| 2 | *IL1-B* | rs1143634 | A/G | Allelic | 98/272 | 27/125 | 4.502 | 1 | 0.03 |
| 2 | *IL1-B* | rs1143634 | A/G | Geno | 18/62/105 | 4/19/53 | 4.032 | 2 | 0.13 |
| 2 | *IL1-B* | rs1143634 | A/G | Dom | 80/105 | 23/53 | 3.799 | 1 | 0.05 |
| 2 | *IL1-B* | rs1143634 | A/G | Rec | 18/167 | 4/72 | 1.392 | **1** | 0.24 |
| 6 | *IL17A* | rs3748067 | T/C | Allelic | 26/388 | 19/139 | 5.207 | 1 | 0.02 |
| 6 | *IL17A* | rs3748067 | T/C | Geno | 1/24/182 | 2/15/62 | 5.177 | 2 | 0.08 |
| 6 | *IL17A* | rs3748067 | T/C | Dom | 25/182 | 17/62 | 4.068 | 1 | 0.04 |
| 6 | *IL17A* | rs3748067 | T/C | Rec | 1/206 | 2/77 | 2.312 | 1 | 0.13 |
| 5 | *IL4* | rs2070874 | T/C | Allelic | 91/339 | 38/116 | 0.8129 | 1 | 0.37 |
| 5 | *IL4* | rs2070874 | T/C | Dom | 78/137 | 32/45 | 0.673 | 1 | 0.41 |
| 5 | *IL4* | rs2070874 | T/C | Rec | 13/202 | 6/71 | 0.284 | 1 | 0.59 |
| 5 | *IL4* | rs2070874 | T/C | Geno | 13/65/137 | 6/26/45 | 0.7462 | 2 | 0.69 |
| 20 | *MMP9* | rs17577 | A/G | Allelic | 56/372 | 43/105 | 19.71 | 1 | **0.000009** |
| 20 | *MMP9* | rs17577 | A/G | Geno | 4/48/162 | 9/25/40 | 19.376 | 2 | **0.00006** |
| 20 | *MMP9* | rs17577 | A/G | Dom | 52/162 | 34/40 | 12.302 | 1 | **0.00045** |
| 20 | *MMP9* | rs17577 | A/G | Rec | 4/210 | 6/65 | 6.821 | **1** | 0.009 |
| 12 | *MYOH1* | rs10850110 | A/G | Allelic | 75/351 | 36/114 | 2.916 | 1 | 0.09 |
| 12 | *MYOH1* | rs10850110 | A/G | Rec | 8/205 | 6/69 | 2.16 | 1 | 0.14 |
| 12 | *MYOH1* | rs10850110 | A/G | Dom | 67/146 | 30/45 | 1.813 | 1 | 0.18 |
| 12 | *MYOH1* | rs10850110 | A/G | Geno | 8/59/146 | 6/24/45 | 3.022 | 2 | 0.22 |
| 19 | *TGFB1* | rs2241715 | A/C | Rec | 22/193 | 9/66 | 0.1819 | 1 | 0.67 |
| 19 | *TGFB1* | rs2241715 | A/C | Allelic | 133/297 | 49/101 | 0.1557 | 1 | 0.69 |
| 19 | *TGFB1* | rs2241715 | A/C | Dom | 111/104 | 40/35 | 0.0648 | 1 | 0.80 |
| 19 | *TGFB1* | rs2241715 | A/C | Geno | 22/89/104 | 9/31/35 | 0.1963 | 2 | 0.91 |
| 11 | *WNT11* | rs1533767 | A/G | Allelic | 90/330 | 27/121 | 0.6789 | 1 | 0.41 |
| 11 | *WNT11* | rs1533767 | A/G | Geno | 13/64/133 | 4/19/51 | 0.751 | 2 | 0.69 |
| 11 | *WNT11* | rs1533767 | A/G | Dom | 77/133 | 23/51 | 0.748 | 1 | 0.39 |
| 11 | *WNT11* | rs1533767 | A/G | Rec | 13/197 | 4/70 | 0.06 | 1 | 0.81 |

*P-values below 0.001 indicate significant associations (shown in bold). CHR=Chromosome, DF=Degrees of Freedom;Test: Allelic, Geno (Genotypic), Dom (Dominant), and Rec (Recessive).

**Results of allelic association tests in Group 2 (adjusted p-values).**

| **CHR** | **Gene** | **SNP** | **Base Pair** | **Allele 1/2** | **Allele 1 Frequency (cases)** | **Allele 2 Frequency (controls)** | **P-value** | **Odds Ratio (95% CI)** |
| --- | --- | --- | --- | --- | --- | --- | --- | --- |
| 20 | *MMP9* | rs17577 | 46014472 | A/G | 0.1308 | 0.2905 | **0.000009** | 0.3676 (0.2338-0.578) |
| 17 | *AXIN2* | rs3923087 | 65553143 | T/C | 0.3168 | 0.4934 | **0.00012** | 0.4761 (0.3253-0.6968) |
| 6 | *IL17A* | rs3748067 | 52190541 | T/C | 0.0628 | 0.1203 | 0.02 | 0.4902 (0.2631-0.9136) |
| 2 | *IL1-B* | rs1143634 | 112832813 | A/G | 0.2649 | 0.1776 | 0.03 | 1.668  (1.037-2.684) |
| 12 | *MYOH1* | rs10850110 | 109386921 | A/G | 0.1761 | 0.24 | 0.09 | 0.6766 (0.4314-1.061) |
| 12 | *AQP5* | rs3736309 | 49964271 | G/A | 0.1435 | 0.09333 | 0.12 | 1.628  (0.8824-3.003) |
| 1 | *BRINP3* | rs1342913 | 190151895 | G/A | 0.4182 | 0.3671 | 0.26 | 1.239  (0.8508-1.806) |
| 5 | *IL4* | rs2070874 | 132674018 | T/C | 0.2116 | 0.2468 | 0.37 | 0.8194 (0.5313-1.264) |
| 11 | *WNT11* | rs1533767 | 76194756 | A/G | 0.2143 | 0.1824 | 0.41 | 1.222  (0.7579-1.971) |
| 19 | *TGFB1* | rs2241715 | 41350981 | A/C | 0.3093 | 0.3267 | 0.69 | 0.923  (0.6201-1.374) |
| 9 | *CA9* | rs2071676 | 35674056 | A/G | 0.3005 | 0.3092 | 0.84 | 0.9595  (0.643-1.432) |
| 3 | *GSK3B* | rs9879992 | 119993874 | G/A | 0.2687 | 0.2714 | 0.95 | 0.9862 (0.6419-1.515) |

*****CHR=Chromosome; gPLINK Allelic Association Test of group 2 (disease phenotype+, TMD+, and PD-) with an adjusted p-value and the application of a Bonferroni correction alpha threshold of 0.001.

**Group 3: Disease phenotype+, TMD-, and PD+**

gPLINK Genotypic Association Test of group 3 with the application of a Bonferroni correction alpha threshold of 0.001 to provide the results of genotypic analysis of study participants presenting with only 1 of the 4 disease phenotypes (asthma, rheumatoid arthritis/autoimmune disease, obesity, or type II diabetes), are negative for TMD, and who are positive for periodontitis (PD).

**Results of genotypic and allelic association tests in Group 3.**

| **CHR** | **Gene** | **SNP** | **Allele 1/2** | **Test** | **Affected** | **Unaffected** | **X^2^** | **DF** | **P-value** |
| --- | --- | --- | --- | --- | --- | --- | --- | --- | --- |
| 12 | *AQP5* | rs3736309 | G/A | Allelic | 110/670 | 14/136 | 2.476 | 1 | 0.12 |
| 12 | *AQP5* | rs3736309 | G/A | Geno | 10/90/290 | 0/14/61 | 2.875 | 2 | 0.24 |
| 12 | *AQP5* | rs3736309 | G/A | Dom | 100/290 | 14/61 | 1.653 | 1 | 0.20 |
| 12 | *AQP5* | rs3736309 | G/A | Rec | 10/380 | 0/75 | 1.965 | 1 | 0.16 |
| 17 | *AXIN2* | rs3923087 | T/C | Allelic | 287/481 | 75/77 | 7.621 | 1 | 0.006 |
| 17 | *AXIN2* | rs3923087 | T/C | Dom | 220/164 | 56/20 | 7.103 | 1 | 0.008 |
| 17 | *AXIN2* | rs3923087 | T/C | Geno | 67/153/164 | 19/37/20 | 7.398 | 2 | 0.02 |
| 17 | *AXIN2* | rs3923087 | T/C | Rec | 67/317 | 19/57 | 2.38 | 1 | 0.12 |
| 1 | *BRINP3* | rs1342913 | G/A | Rec | 64/326 | 9/70 | 1.259 | 1 | 0.26 |
| 1 | *BRINP3* | rs1342913 | G/A | Allelic | 309/471 | 58/100 | 0.466 | 1 | 0.49 |
| 1 | *BRINP3* | rs1342913 | G/A | Geno | 64/181/145 | 9/40/30 | 1.322 | 2 | 0.52 |
| 1 | *BRINP3* | rs1342913 | G/A | Dom | 245/145 | 49/30 | 0.01776 | 1 | 0.89 |
| 9 | *CA9* | rs2071676 | A/G | Dom | 182/214 | 40/36 | 1.139 | 1 | 0.29 |
| 9 | *CA9* | rs2071676 | A/G | Geno | 46/136/214 | 7/33/36 | 2.332 | 2 | 0.31 |
| 9 | *CA9* | rs2071676 | A/G | Rec | 46/350 | 7/69 | 0.3702 | 1 | 0.54 |
| 9 | *CA9* | rs2071676 | A/G | Allelic | 228/564 | 47/105 | 0.2811 | 1 | 0.60 |
| 3 | *GSK3B* | rs9879992 | G/A | Allelic | 222/560 | 38/102 | 0.09103 | 1 | 0.76 |
| 3 | *GSK3B* | rs9879992 | G/A | Rec | 32/359 | 5/65 | 0.08721 | 1 | 0.77 |
| 3 | *GSK3B* | rs9879992 | G/A | Dom | 190/201 | 33/37 | 0.05002 | 1 | 0.82 |
| 3 | *GSK3B* | rs9879992 | G/A | Geno | 32/158/201 | 5/28/37 | 0.1069 | 2 | 0.95 |
| 2 | *IL1-B* | rs1143634 | A/G | Allelic | 188/544 | 27/125 | 4.289 | 1 | 0.04 |
| 2 | *IL1-B* | rs1143634 | A/G | Geno | 43/102/221 | 4/19/53 | 3.567 | 2 | 0.17 |
| 2 | *IL1-B* | rs1143634 | A/G | Dom | 145/221 | 23/53 | 2.337 | 1 | 0.13 |
| 2 | *IL1-B* | rs1143634 | A/G | Rec | 43/323 | 4/72 | 2.786 | 1 | 0.10 |
| 6 | *IL17A* | rs3748067 | T/C | Allelic | 66/736 | 19/139 | 2.357 | 1 | 0.12 |
| 6 | *IL17A* | rs3748067 | T/C | Geno | 4/58/339 | 2/26/92 | 4.023 | 2 | 0.13 |
| 6 | *IL17A* | rs3748067 | T/C | Dom | 62/339 | 17/62 | 1.761 | 1 | 0.18 |
| 6 | *IL17A* | rs3748067 | T/C | Rec | 4/397 | 2/77 | 1.258 | 1 | 0.26 |
| 5 | *IL4* | rs2070874 | T/C | Allelic | 208/574 | 38/116 | 0.2456 | 1 | 0.62 |
| 5 | *IL4* | rs2070874 | T/C | Rec | 37/354 | 6/71 | 0.2152 | 1 | 0.64 |
| 5 | *IL4* | rs2070874 | T/C | Dom | 171/220 | 32/45 | 0.124 | 1 | 0.72 |
| 5 | *IL4* | rs2070874 | T/C | Geno | 37/134/220 | 6/26/45 | 0.254 | 2 | 0.88 |
| 20 | *MMP9* | rs17577 | A/G | Geno | 7/100/277 | 9/25/40 | 23.28 | 2 | **0.000008** |
| 20 | *MMP9* | rs17577 | A/G | Rec | 7/377 | 9/65 | 19.67 | 1 | **0.000009** |
| 20 | *MMP9* | rs17577 | A/G | Allelic | 114/654 | 43/105 | 17.64 | 1 | **0.00002** |
| 20 | *MMP9* | rs17577 | A/G | Dom | 107/277 | 34/40 | 9.519 | 1 | 0.002 |
| 12 | *MYOH1* | rs10850110 | A/G | Allelic | 138/658 | 36/114 | 3.733 | 1 | 0.05 |
| 12 | *MYOH1* | rs10850110 | A/G | Rec | 14/384 | 6/69 | 3.131 | 1 | 0.08 |
| 12 | *MYOH1* | rs10850110 | A/G | Geno | 14/110/274 | 6/24/45 | 4.154 | 2 | 0.13 |
| 12 | *MYOH1* | rs10850110 | A/G | Dom | 124/274 | 30/45 | 2.248 | 1 | 0.13 |
| 19 | *TGFB1* | rs2241715 | A/C | Allelic | 244/544 | 49/101 | 0.17 | 1 | 0.68 |
| 19 | *TGFB1* | rs2241715 | A/C | Dom | 201/193 | 40/35 | 0.1355 | 1 | 0.71 |
| 19 | *TGFB1* | rs2241715 | A/C | Rec | 43/351 | 9/66 | 0.07542 | 1 | 0.78 |
| 19 | *TGFB1* | rs2241715 | A/C | Geno | 43/158/193 | 9/31/35 | 0.1604 | 2 | 0.92 |
| 11 | *WNT11* | rs1533767 | A/G | Allelic | 169/609 | 27/121 | 0.902 | 1 | 0.34 |
| 11 | *WNT11* | rs1533767 | A/G | Geno | 22/125/242 | 4/19/51 | 1.283 | 2 | 0.53 |
| 11 | *WNT11* | rs1533767 | A/G | Dom | 147/242 | 23/51 | 1.204 | 1 | 0.27 |
| 11 | *WNT11* | rs1533767 | A/G | Rec | 22/367 | 4/70 | 0.007 | 1 | 0.93 |

*P-values below 0.001 indicate significant associations (shown in bold). CHR=Chromosome, DF=Degrees of Freedom;Test: Allelic, Geno (Genotypic), Dom (Dominant), and Rec (Recessive).

**Results of allelic association tests in Group 3 (p-value adjustment).**

| **CHR** | **Gene** | **SNP** | **Base Pair** | **Allele 1/2** | **Allele 1 Frequency (cases)** | **Allele 2 Frequency (controls)** | **P-value** | **Odds Ratio (95% CI)** |
| --- | --- | --- | --- | --- | --- | --- | --- | --- |
| 20 | *MMP9* | rs17577 | 46014472 | A/G | 0.1484 | 0.2905 | **0.000026** | 0.4256  (0.2834-0.6393) |
| 17 | *AXIN2* | rs3923087 | 65553143 | T/C | 0.3737 | 0.4934 | 0.006 | 0.6126  (0.4317-0.8693) |
| 2 | *IL1-B* | rs1143634 | 112832813 | A/G | 0.2568 | 0.1776 | 0.04 | 1.6  (1.022-2.504) |
| 12 | *MYOH1* | rs10850110 | 109386921 | A/G | 0.1734 | 0.24 | 0.05 | 0.6641  (0.4376-1.008) |
| 12 | *AQP5* | rs3736309 | 49964271 | G/A | 0.141 | 0.09333 | 0.12 | 1.595  (0.8877-2.865) |
| 6 | *IL17A* | rs3748067 | 52190541 | T/C | 0.08229 | 0.1203 | 0.12 | 0.656  (0.3817-1.127) |
| 11 | *WNT11* | rs1533767 | 76194756 | A/G | 0.2172 | 0.1824 | 0.34 | 1.244  (0.7925-1.952) |
| 1 | *BRINP3* | rs1342913 | 190151895 | G/A | 0.3962 | 0.3671 | 0.49 | 1.131  (0.794-1.611) |
| 9 | *CA9* | rs2071676 | 35674056 | A/G | 0.2879 | 0.3092 | 0.60 | 0.9031  (0.6196-1.316) |
| 5 | *IL4* | rs2070874 | 132674018 | T/C | 0.266 | 0.2468 | 0.62 | 1.106  (0.7421-1.649) |
| 19 | *TGFB1* | rs2241715 | 41350981 | A/C | 0.3096 | 0.3267 | 0.68 | 0.9245  (0.6366-1.343) |
| 3 | *GSK3B* | rs9879992 | 119993874 | G/A | 0.2839 | 0.2714 | 0.76 | 1.064  (0.7107-1.593) |

*CHR=Chromosome; gPLINK Allelic Association Test of group 3 (disease phenotype+, TMD-, and PD+) with an adjusted p-value and the application of a Bonferroni correction alpha threshold of 0.001.

**Group 4: Disease phenotype+, TMD+, and PD+**

gPLINK Genotypic Association Test of group 4 with the application of a Bonferroni correction alpha threshold of 0.001 to provide the results of genotypic analysis of study participants presenting with only 1 of the 4 disease phenotypes (asthma, rheumatoid arthritis/autoimmune disease, obesity, or type II diabetes), are positive for TMD, and who are positive for periodontitis (PD).

**Results of genotypic and allelic association tests in Group 4.**

| **CHR** | **Gene** | **SNP** | **Allele 1/2** | **Test** | **Affected** | **Unaffected** | **X^2^** | **DF** | **P-value** |
| --- | --- | --- | --- | --- | --- | --- | --- | --- | --- |
| 12 | *AQP5* | rs3736309 | G/A | Allelic | 76/386 | 14/136 | 4.572 | 1 | 0.03 |
| 12 | *AQP5* | rs3736309 | G/A | Geno | 12/52/167 | 0/14/61 | 4.905 | 2 | 0.09 |
| 12 | *AQP5* | rs3736309 | G/A | Dom | 64/167 | 14/61 | 2.436 | 1 | 0.12 |
| 12 | *AQP5* | rs3736309 | G/A | Rec | 12/219 | 0/75 | 4.055 | 1 | 0.04 |
| 17 | *AXIN2* | rs3923087 | T/C | Allelic | 147/291 | 75/77 | 11.97 | 1 | **0.0005** |
| 17 | *AXIN2* | rs3923087 | T/C | Dom | 118/101 | 56/20 | 9.145 | 1 | 0.002 |
| 17 | *AXIN2* | rs3923087 | T/C | Geno | 29/89/101 | 19/37/20 | 11.04 | 2 | 0.004 |
| 17 | *AXIN2* | rs3923087 | T/C | Rec | 29/190 | 19/57 | 5.725 | 1 | 0.02 |
| 1 | *BRINP3* | rs1342913 | G/A | Rec | 44/185 | 9/70 | 2.522 | 1 | 0.11 |
| 1 | *BRINP3* | rs1342913 | G/A | Allelic | 194/264 | 58/100 | 1.551 | 1 | 0.21 |
| 1 | *BRINP3* | rs1342913 | G/A | Geno | 44/106/79 | 9/40/30 | 2.523 | 2 | 0.28 |
| 1 | *BRINP3* | rs1342913 | G/A | Dom | 150/79 | 49/30 | 0.3105 | 1 | 0.58 |
| 9 | *CA9* | rs2071676 | A/G | Allelic | 129/331 | 47/105 | 0.4617 | 1 | 0.50 |
| 9 | *CA9* | rs2071676 | A/G | Dom | 112/118 | 40/36 | 0.354 | 1 | 0.55 |
| 9 | *CA9* | rs2071676 | A/G | Rec | 17/213 | 7/69 | 0.2616 | 1 | 0.61 |
| 9 | *CA9* | rs2071676 | A/G | Geno | 17/95/118 | 7/33/36 | 0.4781 | 2 | 0.79 |
| 3 | *GSK3B* | rs9879992 | G/A | Rec | 23/206 | 5/65 | 0.5315 | 1 | 0.47 |
| 3 | *GSK3B* | rs9879992 | G/A | Allelic | 135/323 | 38/102 | 0.2839 | 1 | 0.59 |
| 3 | *GSK3B* | rs9879992 | G/A | Geno | 23/89/117 | 5/28/37 | 0.5318 | 2 | 0.77 |
| 3 | *GSK3B* | rs9879992 | G/A | Dom | 112/117 | 33/37 | 0.0669 | 1 | 0.80 |
| 2 | *IL1-B* | rs1143634 | A/G | Allelic | 114/292 | 27/125 | 6.232 | 1 | 0.01 |
| 2 | *IL1-B* | rs1143634 | A/G | Geno | 30/54/119 | 4/19/53 | 5.271 | 2 | 0.07 |
| 2 | *IL1-B* | rs1143634 | A/G | Dom | 84/119 | 23/53 | 2.89 | 1 | 0.09 |
| 2 | *IL1-B* | rs1143634 | A/G | Rec | 30/173 | 4/72 | 4.678 | 1 | 0.03 |
| 6 | *IL17A* | rs3748067 | T/C | Allelic | 29/433 | 19/139 | 5.447 | 1 | 0.02 |
| 6 | *IL17A* | rs3748067 | T/C | Geno | 0/29/202 | 2/15/62 | 8.12 | 2 | 0.02 |
| 6 | *IL17A* | rs3748067 | T/C | Dom | 29/202 | 17/62 | 3.744 | 1 | 0.05 |
| 6 | *IL17A* | rs3748067 | T/C | Rec | 0/231 | 2/77 | 5.886 | 1 | 0.02 |
| 5 | *IL4* | rs2070874 | T/C | Dom | 74/149 | 32/45 | 1.757 | 1 | 0.19 |
| 5 | *IL4* | rs2070874 | T/C | Allelic | 89/357 | 38/116 | 1.528 | 1 | 0.22 |
| 5 | *IL4* | rs2070874 | T/C | Geno | 15/59/149 | 6/26/45 | 1.793 | 2 | 0.41 |
| 5 | *IL4* | rs2070874 | T/C | Rec | 15/208 | 6/71 | 0.09986 | 1 | 0.75 |
| 20 | *MMP9* | rs17577 | A/G | Rec | 7/216 | 9/65 | 8.875 | 1 | 0.003 |
| 20 | *MMP9* | rs17577 | A/G | Allelic | 79/367 | 43/105 | 8.758 | 1 | 0.003 |
| 20 | *MMP9* | rs17577 | A/G | Geno | 7/65/151 | 9/25/40 | 10.4 | 2 | 0.006 |
| 20 | *MMP9* | rs17577 | A/G | Dom | 72/151 | 34/40 | 4.516 | 1 | 0.03 |
| 12 | *MYOH1* | rs10850110 | A/G | Rec | 9/215 | 6/69 | 1.87 | 1 | 0.17 |
| 12 | *MYOH1* | rs10850110 | A/G | Allelic | 91/357 | 36/114 | 0.9135 | 1 | 0.34 |
| 12 | *MYOH1* | rs10850110 | A/G | Geno | 9/73/142 | 6/24/45 | 1.885 | 2 | 0.39 |
| 12 | *MYOH1* | rs10850110 | A/G | Dom | 82/142 | 30/45 | 0.2761 | 1 | 0.60 |
| 19 | *TGFB1* | rs2241715 | A/C | Rec | 32/197 | 9/66 | 0.1886 | 1 | 0.66 |
| 19 | *TGFB1* | rs2241715 | A/C | Dom | 119/110 | 40/35 | 0.0424 | 1 | 0.84 |
| 19 | *TGFB1* | rs2241715 | A/C | Geno | 32/87/110 | 9/31/35 | 0.3479 | 2 | 0.84 |
| 19 | *TGFB1* | rs2241715 | A/C | Allelic | 151/307 | 49/101 | 0.004692 | 1 | 0.95 |
| 11 | *WNT11* | rs1533767 | A/G | Allelic | 99/343 | 27/121 | 1.14 | 1 | 0.29 |
| 11 | *WNT11* | rs1533767 | A/G | Geno | 7/85/129 | 4/19/51 | 4.326 | 2 | 0.11 |
| 11 | *WNT11* | rs1533767 | A/G | Dom | 92/129 | 23/51 | 2.593 | 1 | 0.11 |
| 11 | *WNT11* | rs1533767 | A/G | Rec | 7/214 | 4/70 | 0.773 | 1 | 0.38 |

*P-values below 0.001 indicate significant associations (shown in bold). CHR=Chromosome, DF=Degrees of Freedom;Test: Allelic, Geno (Genotypic), Dom (Dominant), and Rec (Recessive).

**Results of allelic association tests in Group 4 (p-value adjustment).**

| **CHR** | **Gene** | **SNP** | **Base Pair** | **Allele 1/2** | **Allele 1 Frequency (cases)** | **Allele 2 Frequency (controls)** | **P-value** | **Odds Ratio (95% CI)** |
| --- | --- | --- | --- | --- | --- | --- | --- | --- |
| 17 | *AXIN2* | rs3923087 | 65553143 | T/C | 0.3356 | 0.4934 | **0.0005** | 0.5186  (0.3565-0.7544) |
| 20 | *MMP9* | rs17577 | 46014472 | A/G | 0.1771 | 0.2905 | 0.003 | 0.5256  (0.3419-0.8081) |
| 2 | *IL1-B* | rs1143634 | 112832813 | A/G | 0.2808 | 0.1776 | 0.01 | 1.807  (1.131-2.889) |
| 6 | *IL17A* | rs3748067 | 52190541 | T/C | 0.06277 | 0.1203 | 0.02 | 0.49  (0.2664-0.9011) |
| 12 | *AQP5* | rs3736309 | 49964271 | G/A | 0.1645 | 0.09333 | 0.03 | 1.913  (1.047-3.494) |
| 1 | *BRINP3* | rs1342913 | 190151895 | G/A | 0.4236 | 0.3671 | 0.21 | 1.267  (0.8727-1.839) |
| 5 | *IL4* | rs2070874 | 132674018 | T/C | 0.1996 | 0.2468 | 0.22 | 0.761  (0.4932-1.174) |
| 11 | *WNT11* | rs1533767 | 76194756 | A/G | 0.224 | 0.1824 | 0.29 | 1.293  (0.8058-2.076) |
| 12 | *MYOH1* | rs10850110 | 109386921 | A/G | 0.2031 | 0.24 | 0.34 | 0.8072  (0.52-1.253) |
| 9 | *CA9* | rs2071676 | 35674056 | A/G | 0.2804 | 0.3092 | 0.50 | 0.8707  (0.5838-1.298) |
| 3 | *GSK3B* | rs9879992 | 119993874 | G/A | 0.2948 | 0.2714 | 0.59 | 1.122  (0.7348-1.713) |
| 19 | *TGFB1* | rs2241715 | 41350981 | A/C | 0.3297 | 0.3267 | 0.95 | 1.014  (0.6844-1.502) |

*CHR=Chromosome; gPLINK Allelic Association Test of group 4 (disease phenotype+, TMD+, and PD+) with an adjusted p-value and the application of a Bonferroni correction alpha threshold of 0.001.
